# Tumor-secreted PAI-1 promotes breast cancer metastasis via the induction of adipocyte-derived collagen remodeling

**DOI:** 10.1186/s12964-019-0373-z

**Published:** 2019-06-06

**Authors:** Xiaohui Wei, Sijing Li, Jinyong He, Hongzhi Du, Yang Liu, Wei Yu, Haolin Hu, Lifei Han, Chenfei Wang, Hongyang Li, Xin Shi, Meixiao Zhan, Ligong Lu, Shengtao Yuan, Li Sun

**Affiliations:** 1 Jiangsu Center for Pharmacodynamics Research and Evaluation, China Pharmaceutical University, No. 24, Tongjiaxiang, Nanjing, China; 2 Jiangsu Key laboratory of Drug Screening, China Pharmaceutical University, No.24, Tongjiaxiang, Nanjing, China; 3 School of Pharmacy, Hubei University of Chinese Medicine, Wuhan, China; 4 Breast Disease Center, Zhong-Da Hospital, Southeast University, Nanjing, China; 5 Institute of Dermatology, Chinese Academy of Medical Sciences, Peking Union Medical College, Beijing, China; 6 Department of General Surgery, Zhong-Da Hospital, Southeast University, Nanjing, China; 7 Zhuhai Interventional Medical Center, Zhuhai Precision Medical Center, Zhuhai People’s Hospital, Zhuhai Hospital of Jinan University, Zhuhai, Guangdong China

**Keywords:** Breast Cancer, PAI-1, PLOD2, Microenvironment, Metastasis, Collagen

## Abstract

**Background:**

Breast cancer cells recruit surrounding stromal cells, such as cancer-associated fibroblasts (CAFs), to remodel collagen and promote tumor metastasis. Adipocytes are the most abundant stromal partners in breast tissue, local invasion of breast cancer leads to the proximity of cancer cells and adipocytes, which respond to generate cancer-associated adipocytes (CAAs). These cells exhibit enhanced secretion of extracellular matrix related proteins, including collagens. However, the role of adipocyte-derived collagen on breast cancer progression still remains unclear.

**Methods:**

Adipocytes were cocultured with breast cancer cells for 3D collagen invasion and collagen organization exploration. Breast cancer cells and adipose tissue co- implanted mouse model, clinical breast cancer samples analysis were used to study the crosstalk between adipose and breast cancer cells in vivo. A combination of proteomics, enzyme-linked immunosorbent assay, loss of function assay, qPCR, western blot, database analysis and chromatin immunoprecipitation assays were performed to study the mechanism mediated the activation of PLOD2 in adipocytes.

**Results:**

It was found that CAAs remodeled collagen alignment during crosstalk with breast cancer cells in vitro and in vivo, which further promoted breast cancer metastasis. Tumor-derived PAI-1 was required to activate the expression of the intracellular enzyme procollagen-lysine, 2-oxoglutarate 5-dioxygenase 2 (PLOD2) in CAAs. Pharmacologic blockade of PAI-1 or PLOD2 disrupted the collagen reorganization in CAAs. Mechanistically, it was observed that PI3K/AKT pathway was activated in adipocytes upon co-culturing with breast cancer cells or treatment with recombinant PAI-1, which could promote the translocation of transcription factor FOXP1 into the nucleus and further enhanced the promoter activity of PLOD2 in CAAs. In addition, collagen reorganization at the tumor-adipose periphery, as well as the positive relevance between PAI-1 and PLOD2 in invasive breast carcinoma were confirmed in clinical specimens of breast cancer.

**Conclusion:**

In summary, our findings revealed a new stromal collagen network that favors tumor invasion and metastasis establish between breast cancer cells and surrounding adipocytes at the tumor invasive front, and identified PLOD2 as a therapeutic target for metastatic breast cancer treatment.

**Electronic supplementary material:**

The online version of this article (10.1186/s12964-019-0373-z) contains supplementary material, which is available to authorized users.

## Background

Metastasis is the leading cause of death among breast cancer patients. Tumor metastasis is not only determined by the migration ability of cancer cells themselves, but is also associated with the tumor microenvironment [1]. In the breast cancer microenvironment, the presence of dense collagenous stroma functions as a risk factor for breast carcinoma prognosis [2]. During breast cancer progression, collagen fibers increase in density, accompanied with increased formation of bundled, and aligned collagen. The aligned collagen orientation predicts poor patient survival [3, 4], since tumor cells tend to migrate along aligned stromal collagen fibers in breast cancer [5].

Collagen biogenesis is a complex process that involves a series of post-translational modifications that include lysine hydroxylation [6, 7]. Three lysyl hydroxylase genes (PLOD1, PLOD2 and PLOD3) that encode isoforms of procollagen-lysine, 2-oxyglutarate, 5-dioxygenase have been characterized. PLOD2 specifically hydroxylates lysines in the telopeptides of procollagens to generate aligned and stabilized collagen fibers, while PLOD1 hydroxylates lysine residues of the α-helical or central domain and the substrate of PLOD3 has never been reported. As a major source of collagen, cancer-associated fibroblasts (CAFs) have been shown to generate aligned collagen fibers by activating of PLOD2, and silencing PLOD2 in CAFs results in a major effect on collagen fiber formation and linear organization, which further abrogates collagen-induced directed cell migration [8, 9].

As the predominant component of breast cancer stromal tissue, adipose tissue, functions as a dynamic organ that assists breast tumor cells with growth, invasion and metastasis [10–14]. Growing evidence has demonstrated that breast tumour cells induced changes in the surrounding adipocytes, including delipidation and conversion into a cancer-associated adipocyte (CAA) phenotype [15, 16]. Interestingly, as a consequence of the proximity to invasive cancer cells, CAAs exhibited a “fibroblastic” like morphology, and overexpressed ECM (such as collagen VI, collagen I) or ECM related molecules [17, 18].

As adipocyte-derived fibroblast-like cells have been shown to contribute to the origin of CAFs in the desmoplastic reaction of breast cancers [18]. We questioned the organization feature of CAAs-derived collagen and the underlying regulation mechanism of CAAs-derived collagen reorganization in breast cancer.

In this study, it was demonstrated that breast cancer cells are able to force adipocyte-derived collagen reorganization into bundled and aligned collagen fibers in a PLOD2-dependent way, which further contributes to breast tumor metastasis via both in vitro and in vivo assays (including human tumors). Therefore, our study reveals a potential new novel network of tumor invasion and metastasis that is established between breast cancer cells and surrounding adipocytes, while targeting PLOD2 may be a credible therapeutic strategy for breast cancer metastasis.

## Materials and methods

### Cell culture and reagents

Breast cancer cell lines were obtained from the Cell Bank of the Institute of Biochemistry and Cell biology, Chinese Academy of Sciences (Shanghai, China). Furthermore, all cancer cells were authenticated using Short Tandem Repeat (STR) analysis (Genetic Testing Biotechnology Corporation, Suzhou) to exclude possible contamination. Murine 3 T3-L1 preadipocyte cell line was obtained from ATCC and cultured following the provider’s instructions. Human preadipocyte cells were purchased from ScienCell Research Laboratories and maintained following the provider’s instructions.

### Differentiation assay

Murine 3 T3-L1 preadipocyte cell line was differentiated as previously described [13]. Adipogenesis of human preadipocyte cells was induced using DMEM 10% FBS (Gibico), 1 μM dexamethasone (Sigma), 0.5 mM isobutylmethylxanthine (Sigma), 10 μg/mL insulin (Sigma) and 1 μM rosiglitazone (Sigma) for 72 h, subsequently replaced with DMEM 10% FBS medium containing 10 μg/mL insulin for 72 h, and then maintained in DMEM containing 10% FBS until day 10 of post-adipogenesis induction. The mature adipocytes were identified using bodipy lipid probe (at 2 μg/mL).

### Co-culture and conditioned medium collection

Co-culture assays were conducted as previously described [13]. Briefly, 2 × 10^5^ breast cancer cells (MDA-MB-231, SKBR-3, BT-474 or MDA-MB-468 cells) were seeded into the top chamber of a transwell system (0.4 μM pore size) and cultured either in the presence or absence of adipocytes in the bottom chamber for 72 h. Mature adipocytes cells were cultured alone as control and was evaluated at the same time point. For conditioning of media breast cancer cells were plated in duplicates on 6 well plates at a starting density of 2 × 10^5^ cells/well for 48 h in serum-free medium. Conditioned media were collected and centrifuged for 5 min at 1200 rpm to remove cell debris. Then, 3 mL of the conditioned media were added for 72 h in adipocytes in 6-well plates. Experiments were performed at least 3 times each time in duplicates, and similar results were obtained.

### Cytokine arrays

After 3 days of monoculture or coculture, the cell culture medium was collected, and cytokine concentrations were assayed using a cytokine array kit (RayBiotech, USA). This experiment was performed according to the manufacturer’s instructions and was supported by the Facmacs, Nanjing.

### Enzyme-linked immunosorbent assay (ELISA)

The secretion level of PAI-1 in the cocultured-medium was measured using ELISA kits (RayBiotec, USA) according to the manufacturer’s instructions.

### Transfection and generation of stable cells

shRNAs targeting human PAI-1 (sh1, sh2) as well as a negative control shRNA were received from PPL (China). Lentiviruses were generated with either shRNA or cDNA that had been packaged in 293 T cells using pMD2.G and psPAX2 plasmids. After 48 h, conditioned medium was collected from 293 T cells and used to infect target cells for 72 h. Stable cell lines were generated by selection with puromycin (10 μg/mL).

### Cell adhesion assay

Mature adipocytes co-cultured either with or without breast cancer cells (MDA-MB-231) were placed on the glass coverslips for 3 days. Cells were extracted using BSA containing 25 mmol/L Tris-HCl (pH 7.4), 150 mmol/L sodium chloride, 0.2% Triton X-100 and 20 mmol/L ammonia hydroxide) for 3 min. Cellular debris was carefully washed away with PBS. Then, GFP-tagged MDA-MB-231 cells were seeded onto the cell-free matrices and allowed to adhere to ECMs for 60 min, non-adherent cells were washed off, and adherent cells were taken photographed using phase contrast microscope.

### 3D collagen gel invasion assay

Multicellular aggregates were generated by using the hanging drop method [8]. In brief, MDA-MB-231cells were re-suspended in DMEM medium supplemented with 20% methylcellulose (Sigma) and growth factor–free Matrigel (BD Biosciences), and incubated as droplets (25 μl) containing 1000 cells for 48 h to ensure multicellular aggregation. For collagen invasion assays, the aggregates were washed with medium, and seeded onto rat-tail collagen surface (1.0 mg/ml) containing either adipocytes or CAAs. Aggregate invasion was analyzed at 24 h after seeding, while the medium was refreshed every 3 days, and cell invasion was allowed for up to 7 days until tumor cell invasion was apparent.

### Collagen contraction assay

In brief, type I rat-tail collagen was diluted into concentrations of 3.0 mg/ml and 1 mg/ml. Contraction was conducted in a 3D matrix by embedding adipocytes or CAAs (10^6^ cells per 24 matrices) either with or without MDA-MB-231 cells (10^5^ cells per 24 matrices) into the neutralized collagen I solution. The polymerized matrix was then allowed to contract for up to 10 days in complete medium, which was refreshed every 3 days, and cell contraction ability was evaluated by the area of collagen gel.

### Western blot and RT-PCR assay

Cellular or tissue proteins were extracted and analyzed by western blot. The total cell lysates were extracted from the cells or tissues, adding phosphatase and protease inhibitors. Total proteins (20 μg) were separated via 8–15% SDS-PAGE and transferred onto PVDF membrane (Millipore, USA). Next, the membranes were blocked with 5% BSA and incubated with primary antibodies (dilution in 5% BSA-TBST) for overnight at 4 °C. Then, probed it with secondary antibody for 1 h at room temperature. Subsequently, the expression of the target proteins was detected by Chemiluminescent HRP Substrate (Millipore, USA). The antibodies used in this study were presented in Additional file 1.

The total cellular RNA was isolated using the TRIzol Reagent (Vazyme, Nanjing, China) and reverse transcribed with the HiScript QRT SuperMix for qPCR (Vazyme). The mRNA levels were measured using the SYBR Green master mix (Vazyme), the primers for qPCR assay were showed in Additional file 2: Tables S1 and S2.

### Immunofluorescence and immunohistochemistry assay

Immunohistochemistry and immunofluorescence assays were performed as described previously [19]. 3 T3-L1 cells were seeded on coverslips and was differentiated into adipocytes as previously described [13]. Then adipocytes were either monocultured or cocultured with breast cancer cells for 3 days or treated with PAI-1 recombinant protein for 3 days. Next, cells were fixed with 4% paraformaldehyde for 30 min at room temperature and then permeabilized with 0.3% Triton X-100 in 3% BSA for 15 min at room temperature. Then, 3% bovine serum albumin in PBS was used to block cells for 1 h. Next, cells were incubated with a primary antibody against type I collagen overnight at 4 °C. Cells were incubated with the appropriate secondary antibody and Hoechst for 1 h at room temperature, with extensive washing between every step. Images were captured using a confocal microscope.

Paraffin sections were deparaffinized according to a standard protocol. Antigen retrieval was performed in citrate buffer for 2 min at 100 °C. Sections were blocked with 5% BSA and then incubated with PLOD2 (1:100 diluted) and PAI-1 antibody (1200 diluted) overnight at 4 °C. Sections were then incubated with a biotin-labeled rabbit anti-goat antibody for 30 min at room temperature. Then, sections were visualized with DAB and counterstained with hematoxylin.

### Luciferase reporter assays and the exploration of transcription factors

Luciferase activity was measured using the Dual-Luciferase Reporter Assay System (Promega, Madison, WI, USA) according to the manufacturer’s protocol. The promoter sequence of PLOD2 was obtained via the Ensemble project. The potential transcription factors were predicted by the Genomatix database. Finally, the transcription factors with the highest potential were screened out based on their prediction score (the matrix sim).

### Chromatin immunoprecipitation (ChIP) assay

ChIP assay was performed using a SimpleChIP Plus Enzymatic Chromatin IP Kit (Cell Signaling Technology) according to the manufacturer’s protocol. The final ChIP DNAs were used as templates in qPCR reactions, using primers that encompassed the PLOD2 promoter. The oligonucleotides of primers were showed in Additional file 2: Table S3.

### In vivo fat pad formation and tumor injection

All animal procedures were performed under standard conditions and in accordance with protocols approved by the Experimental Animal Care Commission at China Pharmaceutical University. Mammary adipose tissue (1 ml) was obtained from patients undergoing plastic surgery and implanted subcutaneously into athymic BALB/c nude mice to form a fat pat. MDA-MB-231 cells (1 × 10^6^) were then either injected into the fat pad or alone as a control, while untreated fat pads were also used as a separate control. Tumor growth was directly measured using a caliper. Each group included six mice. After 60 days, the mice were sacrificed and the fat pads, tumors, and fat pads containing tumors were removed and embedded in paraffin after 48 h of fixation in buffered formalin.

### In vivo metastasis assays

Mammary adipose tissue (1 ml) was obtained from patients undergoing plastic surgery and implanted subcutaneously into non-obese diabetic/severe combined immunodeficient (NOD/SCID) mice to form a fat pat. MDA-MB-231(negative control or shPAI-1) cells (1 × 10^6^) were then either injected into the fat pad or injected alone, while untreated fat pads were also used as a separate control. Each group included six mice. After 7 days, the mice injected both adipose and MDA-MB-231 negative control cells were assigned to the negative control group and minoxidil group (8 mice/group, tail vein injection of 0.9% NaCl and 2.5 mg/kg minoxidil at a frequency of once every 2 days, respectively). After 6 weeks, tumor metastasis was assessed by bioluminescent imaging on the Xenogen In Vivo Imaging System (IVIS, Caliper Life Science, Hopkinton, MA). Mice were then sacrificed and lungs were formalin-fixed and paraffin-embedded for hematoxylin and eosin staining. Lung metastases were quantified by human genomic DNA extraction from mouse lungs.

### Database analysis

The correlation of relapse-free survival of breast cancer patients with different gene expressions were analyzed via the Kaplan–Meier plotter (http://kmplot.com/analysis), as described previously [19]. Furthermore, the correlation between two different genes expression in breast cancer tissue was analyzed via the TCGA Research Network (http://cancergenome.nih.gov).

### Human tissue specimens

Patient samples were collected under the guidance of the Biospecimen Repository Core at the Zhongda Hospital (affiliated to Southeast University) with written informed consent of the patients using protocols approved by the Human Subjects Committee at the Southeast University (Approval no: 2015ZDKYSB058). Breast cancer tissues of 16 female patients who were diagnosed with invasive breast carcinoma at Zhongda Hospital, from 2015 to 2017, were collected for IHC analysis in this study. Tissues adjacent to breast cancer tissues were also obtained from these 16 female patients. The clinical pathological characteristics of the 16 patients with breast cancer were showed in Additional file 2: Table S4.

### Statistical analysis

The results are shown as means ± SD. Multiple groups were analyzed using two-way ANOVA followed by the Student-Newman-Keuls multiple comparison test, and comparison between two groups was analyzed using student’s t-test. *p*-value less than 0.05 was considered to be statistically significant.

## Results

### Breast cancer cells promote adipocyte-derived collagen I reorganization in vitro

**Fig. 1:**
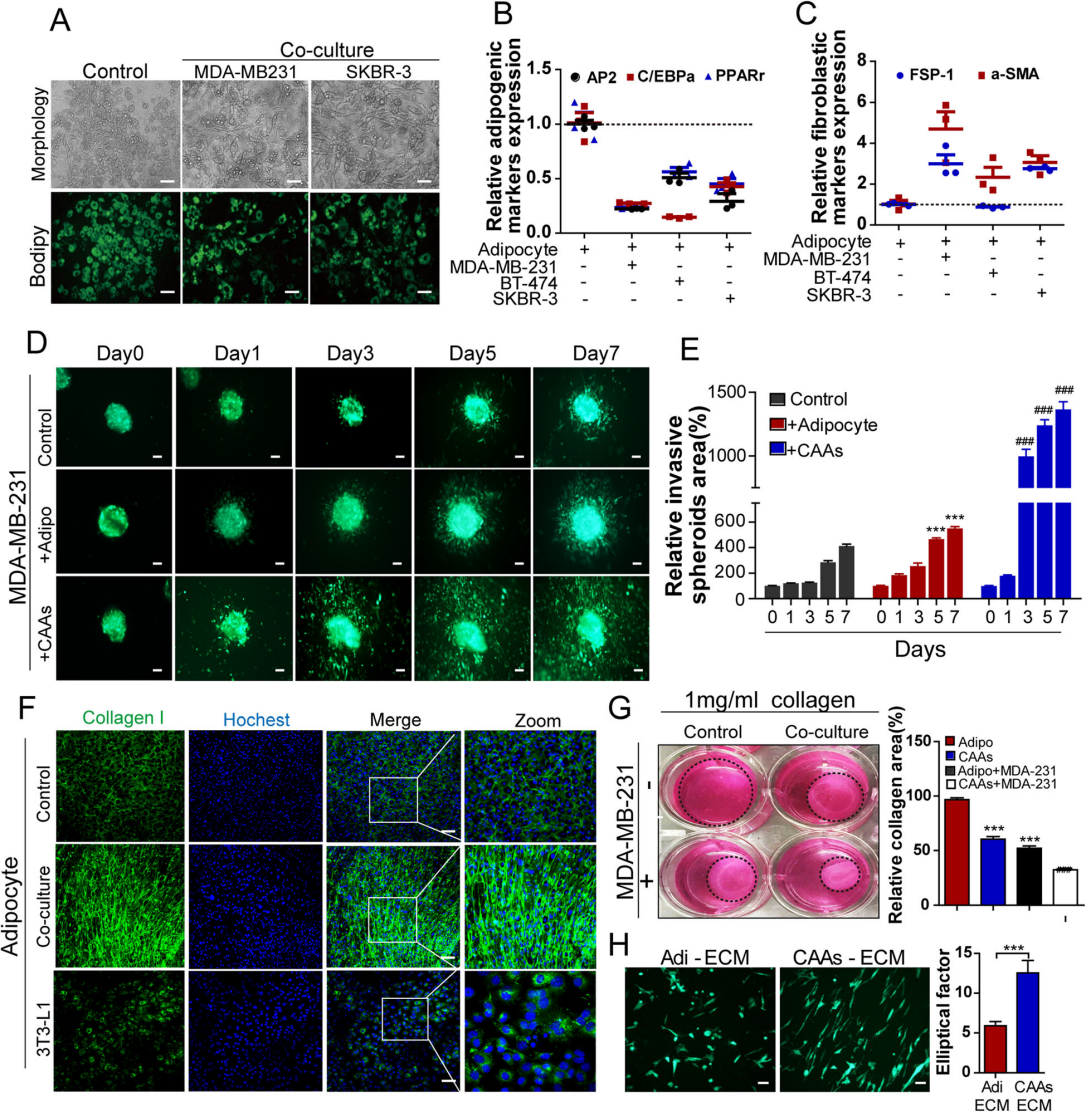
Breast cancer cells promote adipocyte-derived collagen I reorganization in vitro*.* **a** The morphology and bodipy staining of adipocytes alone or cocultured with MDA-MB-231 or SKBR-3 cells. **b** Relative mRNA expression of adipogenic markers, including AP2, C/EBPα and PPARɣ in adipocytes cultured alone or cocultivated with different breast tumor cells (MDA-MB-231, BT-474 or SKBR-3 cells). **c** Relative mRNA expression of fibroblastic markers, including FSP-1 and α-SMA in adipocytes cultured alone or cocultivated with different breast tumor cells (MDA-MB-231, BT-474 or SKBR-3 cells). **d-e** A representative picture of spheroid invasion in a collagen matrix ((left), scale bar: 50 μm, and measurement of the relative invasion capacity at indicated time points (right)). Bars and errors flags represent the means ± SD of three independent experiments, ^*^ indicated adipocytes-derived collagen matrix compared to collagen gel alone at the same time point, ^#^ indicated CAAs- embedded collagen matrix compared with adipocytes- embedded collagen matrix at the same time point. **f** 3 T3-L1 mature adipocytes were grown on coverslips and cultivated in the presence (co-culture) or in the absence (control) of MDA-MB-231 cells for 72 h. After this period, cells were fixed and stained with Collagen I (green) and DAPI (blue). 3 T3-L1 pre - adipocytes served as a control. **g** Photographs showing collagen I matrix (1 mg/ml) contracted in the presence of either adipocytes, CAAs or breast cancer cells. **h** Phase contrast image of GFP-tagged MDA-MB-231 cells, which were either plated on ECM derived from adipocytes or CAAs was shown, the elliptical factor (length/breadth ratio) was calculated for MDA-MB-231 cells plated on ECM derived from adipocytes or CAAs. Bars and errors flags represent the means ± SD of three independent experiments that were classified as statistically significant using student’s t test. Scale bar, 100 μm. ***p < 0.0001versus adipocytes derived ECM

Several studies have reported that cancer cells can induce dedifferentiation of mature adipocytes into fibroblast-like cells. Here, we showed that adipocytes cocultivated in the presence of breast cancer cells with different invasive capacities exhibited a decrease in the number and size of lipid droplets (Fig. 1a), which was accompanied by a dramatic reduction of the adipogenic markers, including AP2, PPAR-γ and C/EBP-α (Fig. 1b). Meanwhile, mature adipocytes changed into a “fibroblastic” shape after co-culture with breast cancer cells, along with an overall increase in fibroblastic markers α-SMA or FSP-1 in cocultivated adipocytes (Fig. 1c). These results were confirmed using western blots (Additional file 3: Figure S1A). In agreement with the results of murine 3 T3-L1 adipocytes, after coculture with breast cancer cells, human ASC adipocytes exhibited a reduction of the adipogenic markers, accompanied by an enhanced expression of fibroblastic markers (Additional file 3: Figure S1B).

In order to assess the role of CAAs in breast cancer invasion, we established an adipocyte-breast cancer cells co-culture collagen gel invasion system. We embedded mature adipocytes or CAAs into collagen gel, and then GFP-labeled breast cancer spheroids were seeded onto the collagen matrix surface. Aggregates of breast cancer cells migrated faster on the CAAs embedded collagen surface, compared with that on adipocytes embedded collagen matrix (Fig. 1d-e).

Functionally activated fibroblasts surrounding tumors are highly contractile and also display increased collagen deposition. Thus, the role of breast cancer on adipocyte-derived collagen fibrillogenesis was analyzed through the comparison of the collagen deposition of mature adipocytes cultured in the presence or absence of breast cancer cells. Immunofluorescence and confocal microscopy revealed that a larger amount of aligned collagen was deposited by CAAs compared to adipocytes (Fig. 1f), consistent results were observed in human ASC adipocytes after cocultured with breast cancer cells (Additional file 3: Figure S1C). Subsequently, it was explored whether CAAs exhibited an enhanced collagen gel contractility capacity. Adipocytes or CAAs were suspended in different concentrations of collagen gel (indicating different stiffinesses) in the presence or absence of breast cancer cells. Actually, the 1 mg/mL collagen gel (approximately 10 kPa) was sufficiently soft for CAAs to contract the gel, while 3 mg/mL collagen gel (approximately 30 kPa) was not able to contract at all (Fig. 1g, Additional file 3: Figure S1D). In addition, CAAs in combination with breast cancer cells could further reduce the collagen areas compared with that of CAAs alone (Fig. 1g). Next, MDA-MB-231 cells were seeded onto cell-free matrix from adipocytes or CAAs and cell morphology was analyzed. Compared with cells plated on adipocytes matrices, cells seeded on CAAs derived ECM exhibited a spindled meschymal morphology (Fig. 1h), which was required for migration and invasion. Altogether, these results suggested that breast cancer cells promoted the adipocyte-derived collagen reorganization, and as a result of this alteration of adipocytes, the migration capacity of breast cancer cells may be further enhanced.

### Activation of PLOD2 in CAAs is responsible for the collagen reorganization in vitro and in vivo

**Fig. 2:**
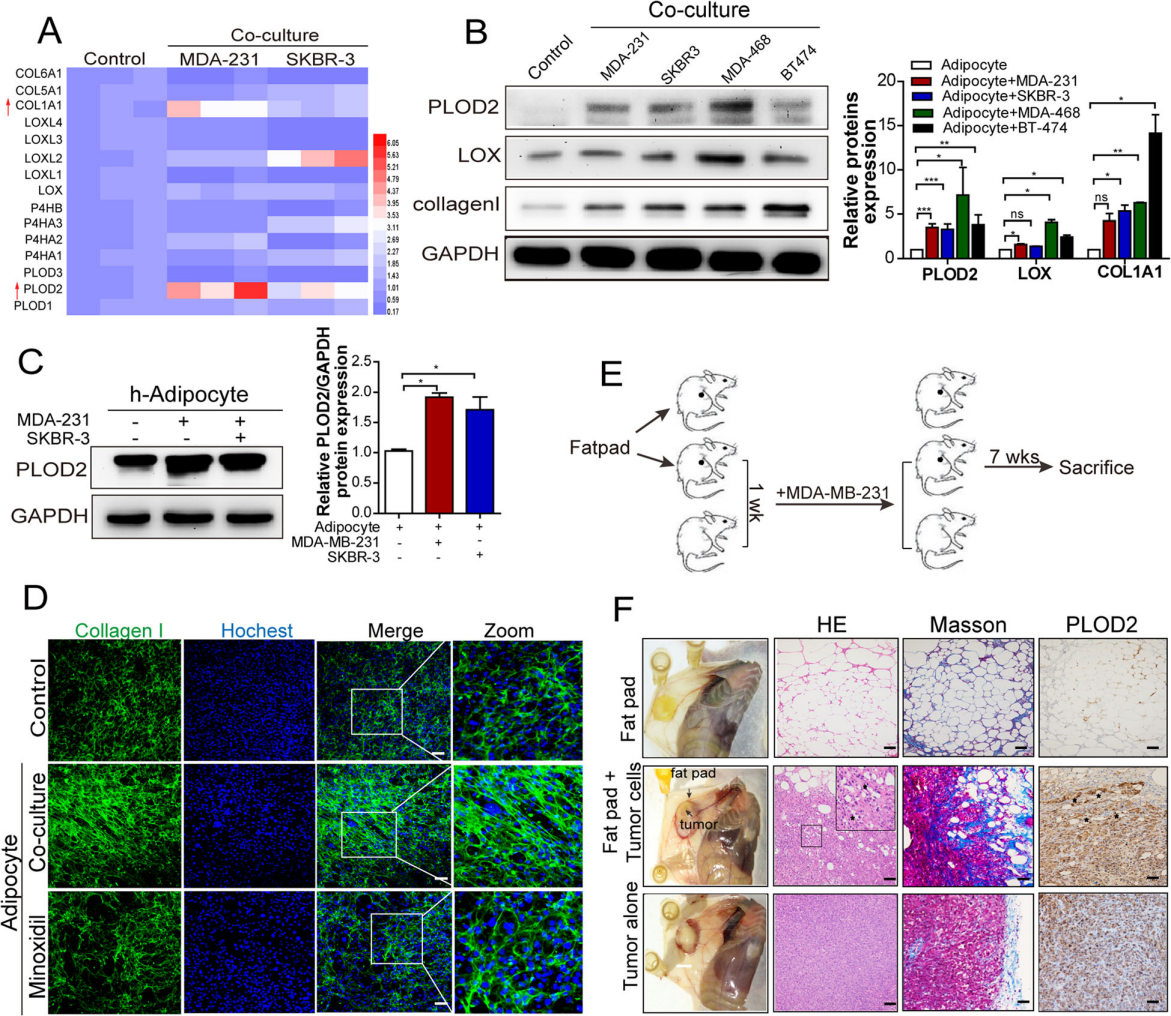
Activation of PLOD2 in CAAs is responsible for the collagen reorganization in vitro and in vivo*.* **a** Relative mRNA expression of collagen modification related genes were measured in the 3 T3-L1 mature adipocytes cultivated in the presence or absence of MDA-MB-231 cells or SKBR-3 cells for 72 h. **b** Protein expression of PLOD2, LOX and collagen I were measured in the 3 T3-L1 adipocytes cultivated in the presence of MDA-MB-231, SKBR-3, MDA-MB-468, BT474 cells or in the absence of breast cancer cells for 72 h, and the bands density of these proteins were further analyzed compared with GAPDH. **c** PLOD2 protein expression was measured in the ASCs adipocytes cultivated in the presence of MDA-MB-231 or SKBR-3 cells or in the absence of breast cancer cells for 72 h, and the band density of PLOD2 was further analyzed compared with GAPDH. **d** Immunofluorescence staining of type I collagen antibody (green) and DAPI (blue) of adipocytes (control), CAAs (cocultured) or CAAs treated with minoxidil (0.5 mM) for 72 h. (**e**) Surgically resected subcutaneous adipose tissue was implanted subcutaneously into athymic BALB/c nude mice, where they were allowed to develop into fat pads after 1 week. MDA-MB-231 cells were then injected into the newly formed fat pads or assayed separately for 8 weeks. **f** Photographs of mice with fat pat alone, fat pat containing tumors and tumors alone after being sacrificed, and HE staining of the different group tumor tissues or fat pats. Masson trichrome and PLOD2 immunohistochemistry stained sections from fat pads alone, tumors grown alone, or tumors grown into the fat pads with collagen stained in blue and the nucleus in red

Post-translational modifications are involved in the architecture of collagen, in particular the hydroxylation and oxidation lysyl residues. To determine the regulatory mechanism of collagen reorganization in adipocytes, we analyzed the expression of collagen-related modification enzymes, including prolyl hydroxylase, lysyl hydroxylase and lysyl oxidase expression in mature 3 T3-L1 adipocytes cultured either with or without breast cancer cells. qPCR array showed that adipocytes exhibited a significant increase in PLOD2 and collagen I expression when cocultivated with breast tumor cells (Fig. 2a). Immunoblot assay further confirmed that different breast cancer cells dramatically induced PLOD2 expression in adipocytes, while exerted little effect on LOX protein expression (Fig. 2b). Consistently, cocultured with breast cancer cells led to a dramatical upregulation of PLOD2 expression in human ASC adipocytes (Fig. 2c). In order to determine whether PLOD2 is involved in CAAs-derived collagen remodeling, the PLOD2 pharmacological inhibitor minoxidil was used to inhibit the PLOD2 expression in CAAs, and immunofluorescence staining showed that linear collagen became disorganized after the minoxidil treatment (Fig. 2d).

Next, whether the peritumoral adipocytes exhibited similar alteration in vivo was evaluated. Mammary adipose tissue was implanted subcutaneously into athymic BALB/c nude mice to form fat pats. MDA-MB-231 cells were then injected into the fat pads or injected alone as a control, while untreated fat pads also provided a separate control (Fig. 2e). In the case of tumors combined with the fat pads, tumor cells invaded into the adipose tissue, and adipose tissue at the tumor periphery was composed of adipocytes with smaller size and elongated morphology (Fig. 2f, left). Staining of the tissue sections with masson trichrome revealed a significant amount of bundled collagen fibrils in the tumors grown with fat pads, whereas fewer collagen fibrils were seen in tumor grown alone (Fig. 2f, middle). Moreover, immunohistochemistry staining showed that PLOD2 was higher expression in the adipose tissues at the tumor periphery than adipose tissues grown alone (Fig. 2f, right). Taken together, these data demonstrated that CAAs-derived aligned type I collagen deposition is mediated by the activation of PLOD2 in CAAs.

### PAI-1, rather than IL-6, mediates the activation of PLOD2 and promotes collagen alignment in CAAs

**Fig. 3:**
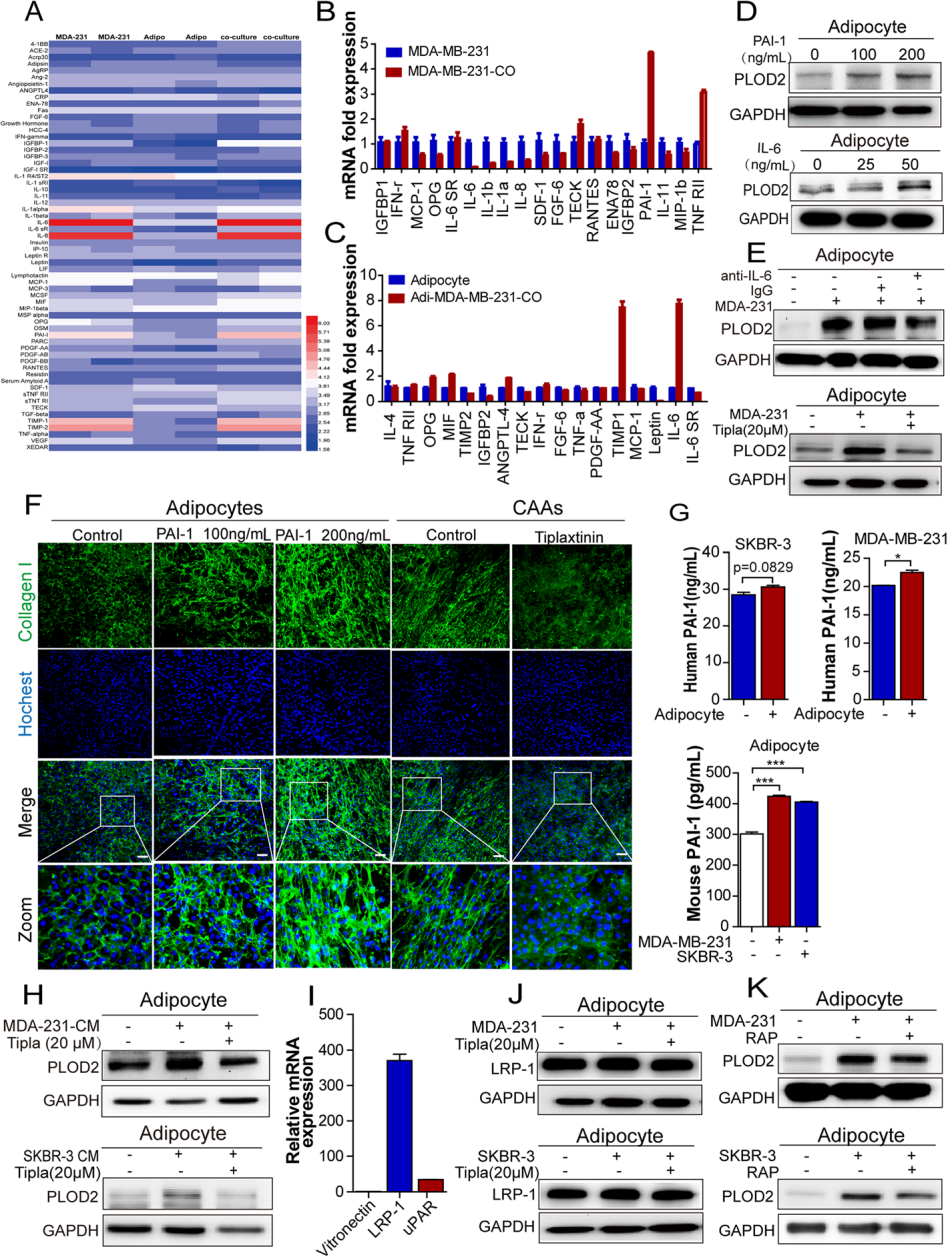
PAI-1, rather than IL-6 induces PLOD2 activation and promotion of collagen alignment in CAAs. **a** Heatmap representing the intensities of each spot of cytokines in the medium. MDA-MB-231 cells were co-cultured with adipocytes for 3 days. Then medium from co-cultured group, cancer alone and adipocytes alone group were collected, and membrane based assays were used to detect cytokines in the medium. **b**, **c**) mRNA expression of cytokines in MDA-MB-231 cultured alone or with adipocytes (MDA-MB-231-CO), and adipocytes cultured alone or with MDA-MB-231for 72 h by qPCR. Data was normalized to 1 for monocultures using GAPDH as an internal control. **d** Expression of PLOD2 fed with indicated PAI-1and IL-6 recombinant proteins. **e** Immunoblot assay of adipocytes cultured alone or cocultured with MDA-MB-231 cells treated with 5 μg/μl IL-6 neutralizing antibody or tiplaxtinin (20 μM) for PLOD2 expression. **f** Immunofluorescence staining of type I collagen antibody (green) and DAPI (blue) of adipocytes (control), CAAs (cocultured) or CAAs treated with tiplaxtinin (20 μM) for 72 h, or adipocytes treated with PAI-1 recombinant protein (100 ng/ml, 200 ng/ml) for 72 h. **g** PAI-1 concentrations in the medium conditioned by 72 h of culturing of MDA-MB-231or SKBR-3, adipocytes, or adipocytes-cancer cells cocultured were analyzed using Elisa. **h** Western blot analysis of PLOD2 in cancer cells (MDA-MB-231 and SKBR-3) of the conditioned medium (CM) – treated adipocytes. **i** PAI-1 receptors of adipocytes were analyzed using qPCR assay. **j** Analysis of protein expression of LRP-1 was done using western blots with extracts obtained from adipocytes cocultivated in the presence or absence of tumor cells, which were treated with tiplaxtinin for 72 h. **k** Analysis of protein expression of PLOD2 from adipocytes cocultivated in the presence or absence of tumor cells was done using western blots, which were treated with RAP (10 μg/ml) for 72 h

Based on the in vitro co-culture system, the effects of breast cancer are most likely mediated by paracrine or autocrine of soluble factors. To explore potential factors of the co-culture system, the medium of mature adipocytes or MDA-MB-231 cells cocultured either with or without adipocytes were exposed to cytokine antibody microarrays. Among the 62 proteins, the mostly secreted cytokines in the co-culture system were IL-6, IL-8, PAI-1, TIMP1, and TIMP2 (Fig. 3a). Next, the top 20 cytokines gene expressions that were markedly altered in both MDA-MB-231 cells cocultured either with or without adipocytes (MDA-MB-231 or MDA-MB-231-CO) and adipocytes cocultured either with or without MDA-MB-231 cells (Adipocyte or Adi-MDA-MB-231-co) were further verified. PAI-1 expression increased 5-fold in MDA-MB-231 cells when cocultured with adipocytes, while IL-6 gene expression was upregulated at approximately 8-fold in CAAs (Fig. 3b-c).

To further verify the importance of IL-6 and PAI-1 in the regulation of PLOD2, we first supplemented mature adipocytes with recombinant PAI-1 and IL-6, and observed that PLOD2 expression was significantly upregulated in adipocytes after stimulated with PAI-1 and IL-6 recombinant proteins (Fig. 3d). Interestingly, PLOD2 expression was efficiently reduced to the level found in mature adipocytes after treatment with the PAI-1 inhibitor (tiplaxtinin) (Fig. 3e and Additional file 4: Figure S2B), whereas the IL-6 neutralizing antibody exerted only a slight effect on PLOD2 expression in CAAs (Fig. 3e and Additional file 4: Figure S2A). In addition, tiplaxtinin treatment significantly abrogated the well-organized collagen deposition by CAAs, while stimulation with PAI-1 increased the amount of linear collagen compared with that of adipocytes alone (Fig. 3f).

PAI-1 is one of the most important adipokines. Simultaneously, PAI-1 exerts various effects on multiple aspects of cancer progression, including migration, invasion and apoptosis [20–23]. Thus, we evaluated whether PAI-1 was secreted by adipocytes or paracrinically secreted by breast cancer cells using ELISA assays. Interestingly, both adipocytes and breast cancer cells secreted more PAI-1 after coculture, however breast cancer cells secreted much more PAI-1 than adipocytes (> 5 folds) (Fig. 3g). Furthermore, the conditioned medium of cancer cells (MDA-231-CM or SKBR3-CM) significantly stimulated the PLOD2 expression in adipocytes, while the PAI-1 inhibitor reversed the promotion effect (Fig. 3h). In addition, after knockdown of PAI-1 in MDA-MB-231 cells, the upregulation effect of PLOD2 expression was abrogated in CAAs (Additional file 4: Figure S2E-F).

PAI-1 has been shown to mediate signal transduction effect by binding to membrane receptors, including uPAR, vitronectin and LRP1. Here, our qPCR data showed the extremely high level of LRP-1 in adipocytes, while vitronectin was only slightly expressed in adipocytes and uPAR was moderately expressed (Fig. 3i). We further tested the alteration of LRP-1 in CAAs using immunoblots, which indicated that LRP-1 was highly expressed in both adipocytes and CAAs (Fig. 3j). Furthermore, adipocytes stimulation with high concentrations of PAI-1(200 ng/ml) resulted in the upregulation of LRP-1 (Additional file 4: Figure S2G). In order to verify the role of LRP-1 in mediating activation of PLOD2 in CAAs, we added the neutralizing antibody of LRP-1, RAP, to inhibit the role of LRP-1, and the results showed RAP abrogated the PLOD2 expression in CAAs (Fig. 3k).

Taken together, these results indicate that paracrinically secreted PAI-1, rather than IL-6 promotes PLOD2 activation in CAAs in a LRP-1 dependent manner.

### Disruption of PLOD2 or knockdown of PAI-1 abrogates linearized collagen and tumor metastasis in vivo

**Fig. 4:**
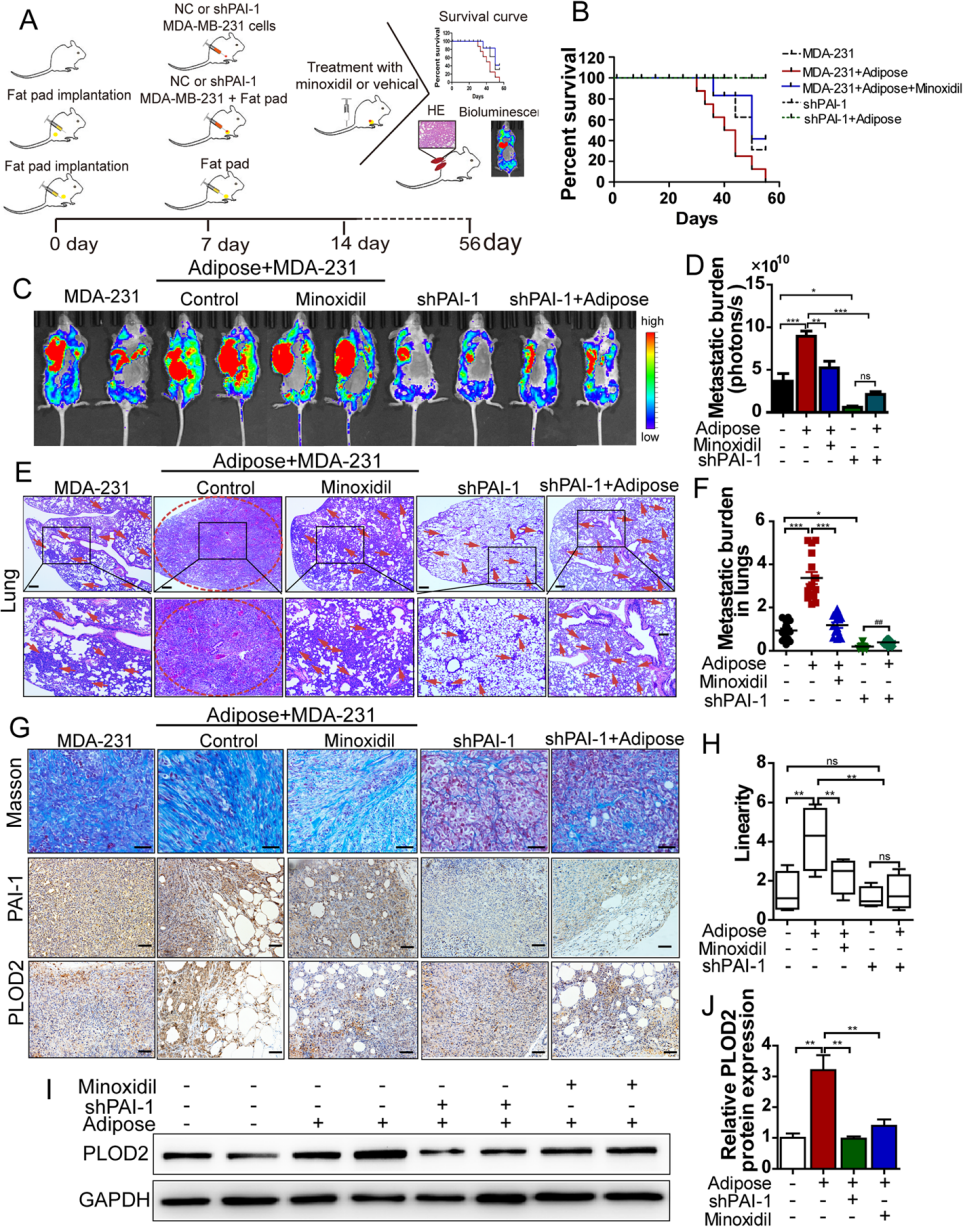
Disruption of PLOD2 or knockdown of PAI-1 abrogates linearized collagen and tumor metastasis in vivo. **a** Schematic diagram of the development of the breast cancer metastasis model. **b** Kaplan-Meier survival analysis for the mice. **c,d** Representative images and quantification bioluminescence of breast tumor bearing mice treated with minoxidil (2.5 mg/kg). Metastatic burden was detected by bioluminescent imaging (BLI) 56 days after injection. **e** The metastatic nodes in the lungs were detected by hematoxylin and eosin (H&E) staining. Arrows indicate metastatic colonization. Scale bars, 100 μm. **f** Human DNA content in mouse lungs determined by qPCR (*n* = 6). **g** The collagen deposition and PAI-1, PLOD2 expression in tumors were detected by Masson trichrome staining and immunohistochemistry staining, respectively. **h** The degree of fibrillar organization was evaluated by a pathologist. The linearity score (from 0 to10) indicates the degree of fibrillar organization. Higher score suggested greater fibrillar organization. **i** Immunoblot analysis of PLOD2 expression in tumor tissues from MDA-MB-231 injected alone, or co-injection MDA-MB-231 cells with adipose, with or without minoxidil treatment, or silencing of PAI-1 in the MDA-MB-231 cells co-injection with adipose tissues. **j** The relative PLOD2 expression at protein levels were analyzed by using GAPDH as the loading control (*n* = 4 in different groups). The *P*-values < 0.05 were considered statistically significant for all tests.

Given that our above results showed that tumor-derived PAI-1 stimulated adipocyte-derived collagen reorganization via upregulation of PLOD2 expression. It was worthy to investigate the role of PLOD2-mediated structural changes of adipose-derived collagen on the regulation of distal organ seeding and growth of metastatic tumor cells in vivo. We conducted in vivo adipose tissue and MDA-MB-231 cells co-injected assay. After the formation of fat pad, MDA-MB-231 (shPAI-1 or negative control) cells were injected into the fat pad (Fig. 4a). After 8 weeks, compared with MDA-MB-231 cells alone, co-injected of MDA-MB-231 cells and adipose tissue increased the metastatic lesions, especially the number of metastatic nodes in lungs, and decreased the survival time of mice (Fig. 4b-f). However, inhibition of PLOD2 via minoxidil improved the quality of life compared with the control groups, while the metastasis nodes were markedly reduced in lungs when PLOD2 was inhibited (Fig. 4b-f).

In addition, knockdown of PAI-1 markedly decreased the metastatic lesions and prolong the survival of mice when co-injected with adipose tissue compared with control group (Fig. 4b-f). Consistent with these findings, masson’s trichrome staining of tumors showed that the collagen was more linearized in control group than MDA-MB-231 cells injected alone, minoxidil treatment significantly reduced the linear collagen compared with control group (Fig. 4g and h). Moreover, knockdown of PAI-1 markedly abrogated the amount of linear collagen compared with the control group (Fig. 4g and h), suggesting that the aligned “highway” for cancer cell migration was reversed. Furthermore, immunohistochemistry staining and the immunoblotting results showed that PLOD2 expression was upregulated when MDA-MB-231 cells co-injection with adipose tissue compared to MDA-MB-231 cells injection alone, while silencing of PAI-1 or treatment with minoxidil both reduced the PLOD2 expression in tumors (Fig. 4g, i, j).

Thus, PLOD2 mediated cancer-associated adipose caused structural changes of collagen to promote breast cancer metastasis. Collectively, our results inferred that PLOD2 inhibitor attenuated breast cancer metastasis, at least in part through hampering the collagen structural.

### PI3K/AKT signaling pathway rather than JAK/STAT3 signaling pathway contributes to PLOD2 activation in CAAs

**Fig. 5:**
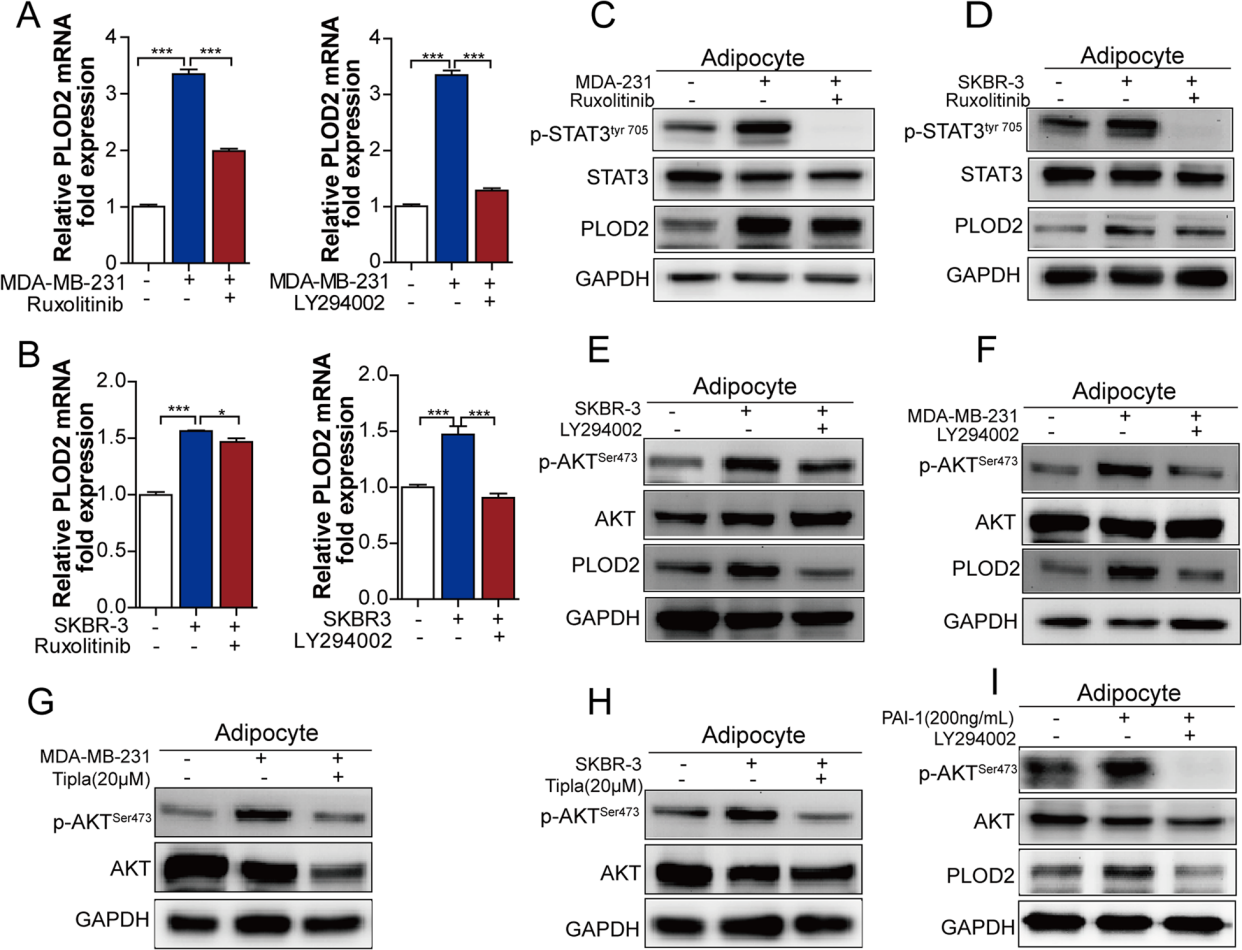
PI3K/AKT signaling pathway, rather than JAK/STAT3 signaling pathway that contributes to PLOD2 activation in CAAs. **a,b** Adipocytes were cultivated in the presence or in the absence of breast tumor cells (MDA-MB-231 or SKBR-3), and in the presence or absence of JAK inhibitor or PI3K inhibitor. In these adipocytes, the expression of PLOD2 at mRNA level was measured by qPCR assay. **c,d** Analysis of phosphorylation (tyr705) STAT3, total STAT3 and PLOD2 protein expression was done using western blot analysis with extracts from mature adipocytes cultivated in the presence or in the absence of breast cancer cells (MDA-MB-231 or SKBR-3), and in the presence or absence of JAK inhibitor (ruxolitinib). **e,f** Analysis of phosphorylation (tyr705) STAT3, total STAT3 and PLOD2 protein expression was done using western blot analysis with extracts from mature adipocytes cultivated in the presence or in the absence of breast cancer cells (MDA-MB-231 or SKBR-3) in the presence or absence of PI3K inhibitor (LY294002). **g,h** Western blot analysis of phosphorylation (ser473) of AKT and total AKT of adipocytes in the presence or absence of breast cancer cells (MDA-MB-231(**g**) or SKBR-3(**h**)), which treated with tiplaxtinin in the cocultured system. **i** Analysis of phosphorylation (ser473) of AKT and total AKT of adipocytes in the presence or absence of PAI-1(200 ng/ml), which was treated with combination of LY294002

PAI-1 has been shown to affect several signaling molecules that regulate cellular processes via the PI3K/AKT and JAK/STAT1/3 signaling pathways [24, 25]. Thus, PI3K inhibitor or JAK inhibitor was added into the coculture system, and the results showed that inhibition of JAK activity and PI3K activity both alleviated PLOD2 mRNA expression in CAAs (Fig. 5a), while the protein level of PLOD2 in CAAs was unaffected by JAK inhibitor (Fig. 5c-d), but the PI3K inhibitor significantly attenuated protein level of PLOD2 expression in CAAs (Fig. 5b, e-f). These results indicated that PI3K/AKT pathway might be predominantly involved in regulating PLOD2 expression in CAAs.

To further validate whether PI3K/AKT is activated by PAI-1 in CAAs, tiplaxtinin was added to the coculture model, it showed that tiplaxtinin reduced AKT phosphorylation in CAAs (Fig. 5g-h). Meanwhile, recombinant PAI-1 protein activated AKT phosphorylation and PLOD2 in adipocytes, while inhibition of the PI3K/AKT pathway impaired the increase of PLOD2 caused by recombinant PAI-1 protein (Fig. 5i). Therefore, our results showed that the PI3K/AKT signaling pathway contributes to PAI-1-induced PLOD2 activation in CAAs rather than the JAK/STAT3 signaling pathway.

### Transcription factor FOXP1 has a role in mediating PLOD2 activation in CAAs

**Fig. 6:**
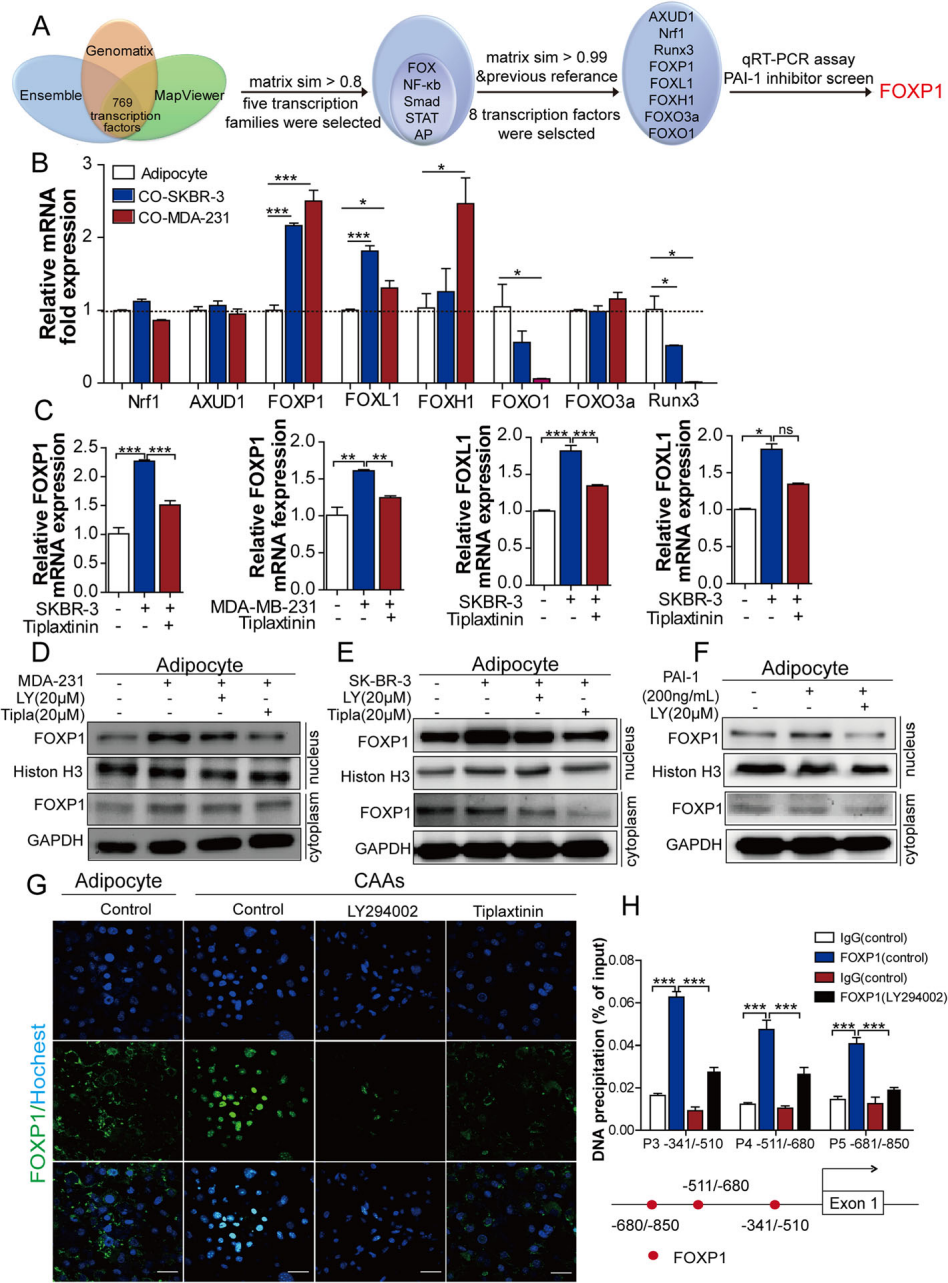
FOXP1 acts as a transcription factor of PLOD2 in CAAs. **a** FOXP1 was selected as the potential transcription factor of PLOD2 based on a series of databases and qPCR verification. First, the promoter sequence of PLOD2 was obtained via the Ensemble project and Mapviewer. Then, 769 potential transcription factors were predicted using the Genomatix database, and eight of the transcription factors with highest potential were screened out based on the prediction score (the matrix sim). Finally, based on qPCR analysis and inhibitor treatment, FOXP1 was identified as a potential transcription factor. **b** The gene expression of eight potential transcription factors were evaluated in adipocytes cultivated in the presence or in the absence of tumor cells (MDA-MB-231 and SKBR-3 cells) for 72 h. **c** The expression of FOXP1 and FOXL1 were evaluated in adipocytes cultivated in the presence or in the absence of tumor cells (MDA-MB-231 and SKBR-3 cells) with tumor cells in the presence of tiplaxitin for 72 h. **d,e** The PI3K/AKT signaling pathway inhibitor (LY294002) and tiplaxitin inhibited the nuclear translocation of FOXP1 through the separation of the cytoplasm and nuclear proteins of adipocytes cultivated in the presence or in the absence of tumor cells (MDA-MB-231 and SKBR-3 cells) for 72 h. **f** The PI3K/AKT signaling pathway inhibitor (LY294002) inhibited the nuclear translocation of FOXP1 through the separation of the cytoplasm and nuclear proteins of adipocytes cultivated in the presence or in the absence of PAI-1 (200 ng/ml). **g** Adipocytes grown on coverlips cultured in the presence or in the absence of tumor cells with tumor cells in the presence of tiplaxitin or LY294002 for 72 h. Cells were fixed and stained with the FOXP1 antibody (green), nuclei were stained with Hochest (blue). Scale bars, 100 μm. **h** Three putative bind regions of FOXP1 upstream of the transcription start site (TSS) of PLOD2 gene were found using ChIP assay. The PI3K/AKT signaling pathway inhibitor (LY294002) was used to inhibit the binding regions of the PLOD2 gene. Precipitated DNA fragments in ChIP assays were examined using qPCR. Immunogobulin G (IgG) was used as a negative control, schematic representation of FOXP1-binding sites upstream of the TSS of PLOD2 using ChIP assay. The *P*-values < 0.05 was considered statistically significant for all tests

The results of our study showed the activation of PI3K/AKT pathway increased PLOD2 expression. Further dual-luciferase assays demonstrated PAI-1 could positively regulate the transcription of PLOD2, as tiplaxtinin inhibited the promoter activity of PLOD2 (Additional file 5: Figure S3A). These results indicate that a missing link exists between PI3K/AKT and PLOD2 transcription. To solve this question, we next sought to find the transcription factors of PLOD2 that are regulated by the PI3K/AKT signaling pathway. First, the promoter sequence of PLOD2 was obtained via the Ensemble project. Then, the potential transcription factors were predicted using the Genomatix database and MapViewer. Finally, the eight most potential transcription factors with highest potential were identified based on the prediction scores (matrix sim > 0.99) (Fig. 6a). Thereby, expression levels of these transcription factors were validated in adipocytes and CAAs, and it was found that the mRNA level of two transcription factors, FOXP1 and FOXL1 were enriched more in CAAs than adipocytes (Fig. 6b). Subsequently, these two transcription factors were detected in CAAs when treated either with or without tiplaxtinin, with the result that only FOXP1 was upregulated in CAAs, and this upregulation was reversed by tiplaxtinin at both transcription and translation levels (Fig. 6c and Additional file 5: Figure S3B-C).

As a transcriptional factor, the potential nuclear translocation of FOXP1 in the activation of PLOD2 was further investigated. As shown in Fig. 6d and e, the accumulation of FOXP1 in the nucleus of adipocytes was dramatically enhanced when cocultured with MDA-MB-231 cells or SKBR-3 cells, while this phenomenon was reversed by the PI3K/AKT inhibitor and tiplaxtinin. Moreover, to further determine whether PAI-1 mediates the translocation of FOXP1, we measured the protein levels of FOXP1 in nuclear and cytosolic fractions after treatment with recombinant PAI-1 protein. As shown in Fig. 5f, PAI-1 specifically induced nuclear translocation of FOXP1, while this effect was notably inhibited by the PI3K/AKT inhibitor. Similar results were obtained by immunofluorescence staining, with FOXP1 significantly accumulated in nucleus of adipocytes after cocultivated with breast cancer cells, while the PI3K/AKT inhibitor or tiplaxtinin decreased the distribution of FOXP1 in the nucleus (Fig. 6g).

To confirm the binding sites of FOXP1 to promoter and enhancer regions of PLOD2, CHIP assay was performed using twelve pairs of primers covering these promoter regions. The results showed that FOXP1 bound to 3 putative binding regions including − 341/− 510, − 511/− 680 and − 681/− 850, while blocking of the PI3K/AKT signaling pathway could significantly reduce the promoter activity of FOXP1 binding sites (Fig. 6h). These results collectively demonstrate that FOXP1 is able to regulate PLOD2 transcription by directly binding to the PLOD2 promoter.

### Clinical relevance of PAI-1 and PLOD2 around mammary tumor-adipose periphery

**Fig. 7:**
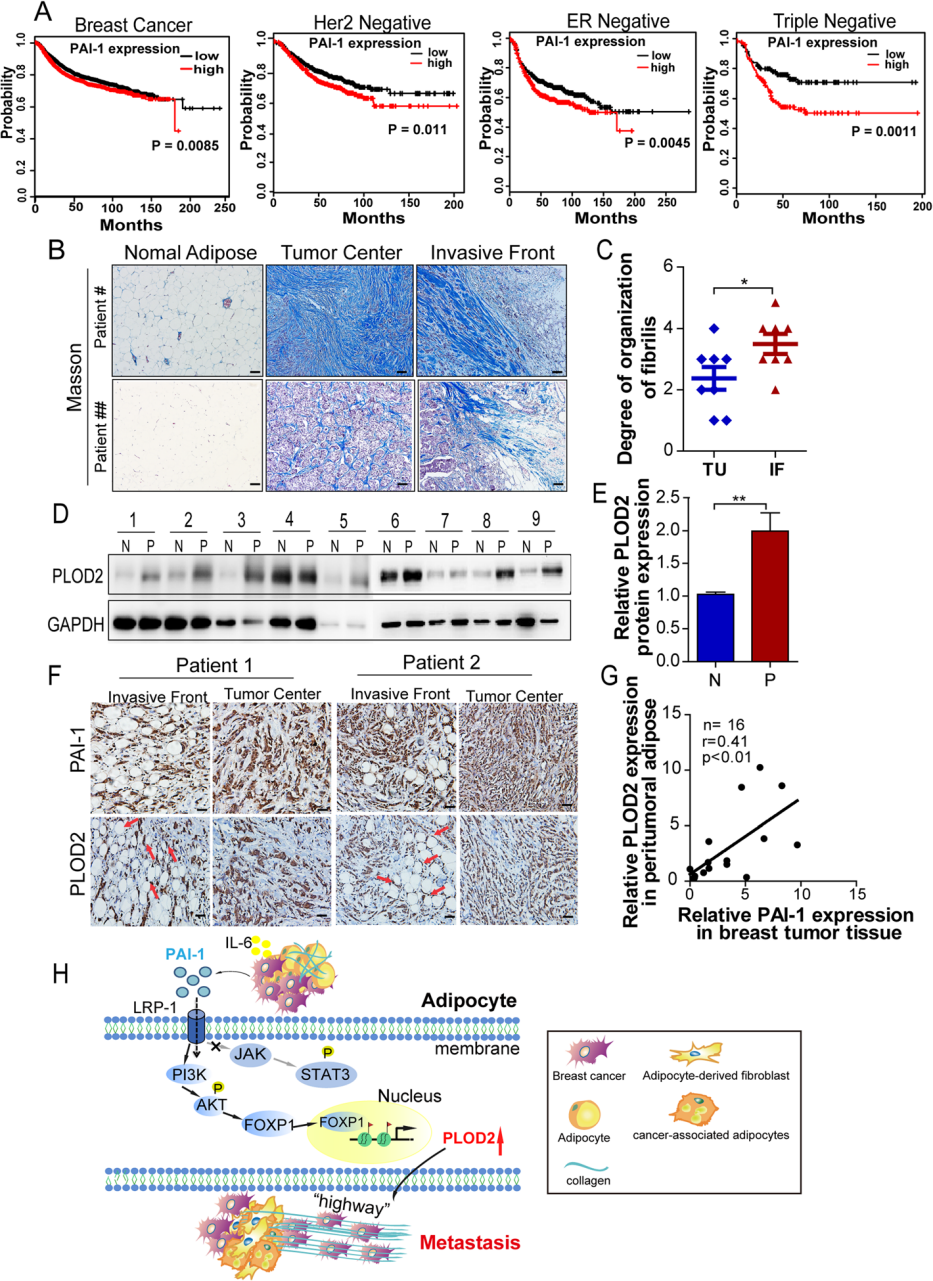
Clinical relevance of PAI-1 and PLOD2 in mammary tumor-adipose periphery. **a** Kaplan–Meier (KM) analysis of relapse-free survival of breast cancer patients from KM plotter (http://kmplot.com/analysis). PAI-1 in breast cancer patients (*n* = 3971), in HER2 negative breast cancer patients (*n* = 800), in ER negative breast carcinoma patients (*n* = 801), and in triple negative breast carcinoma patients (*n* = 255). **b** Masson trichrome stained of invasive breast tumors, sections from far away normal mammary adipose tissue, tumors center, and the invasive front of tumors, collagen fibrillar was stained in blue and the nucleus in red (original magnification × 100). **c** The degree of collagen fibrillar organization was evaluated by a pathologist. The score (from 0 to 6) indicates the degree of fibrillar organization. A higher score indicated greater fibrillar organization. The *P-values < 0.05 were considered statistically significant for all tests. **d** Western blot showing expression of PLOD2 in normal adipose tissue (N) and the corresponding peri-tumor adipose tissue (P) from nine invasive breast cancer patients. **e** The relative PLOD2 expression at protein levels was analyzed by using GAPDH as the loading control (*n* = 9). **P-values < 0.01 were considered statistically significant for all tests. **f** Histological examination of PAI-1 and PLOD2 was visualized (brown) in adipocytes located adjacent to invading cancer cells (Invasive front) and the tumor center. **g** Correlation analysis of PLOD2 in peritumoral adipose tissue and PAI-1 in breast cancer patients. **h** Schematic diagrams show the heterotypic interaction between breast cancer cells and adipocytes in the breast cancer microenvironment. Tumor-derived PAI-1 promotes adipocyte-derived collagen reorganization in a paracrine manner. PAI-1 secreted from breast cancer cells can promote PLOD2 expression in adipocytes via activating the PI3K/AKT-FOXP1 axis. Activation of PLOD2 induces the linear organization of adipocyte-derived collagen, which leads to tumor cells metastasis

More importantly, this crosstalk was further confirmed via the analysis of clinical samples from breast cancer patients. The prognosis role of PAI-1was evaluated in different subtypes of breast cancer using the Kaplan-Meier plotter (http://kmplot.com/analysis/), the results indicated that high expression of PAI-1 was correlated with poor prognosis of breast cancer, especially in HER2 negative cancers, ER negative cancers and triple negative cancers (Fig. 7a). Masson trichrome staining was used to further detect the collagen deposition and organization in clinical samples, and the results showed that collagen exhibits a linear alignment at the tumor periphery around adipose tissues (Fig. 7b-c). Next, the PLOD2 expression was analyzed in clinical mammary adipose samples using western blot, which showed that PLOD2 expression was significantly elevated in 7/9(77.8%) of the tumor-adjacent adipose tissues compared with that of normal adipose tissues with the exception of case no.4 and 7(Fig. 7d-e).

In order to study the clinical relevance between PAI-1 in breast tumor tissues and PLOD2 expression in peripheral adipose tissues, 16 pairs of samples in each group were examined (Additional file 2: Table S4 for the clinical characteristics of the patients). PLOD2 expression in invasive front of adipose tissues and PAI-1 expression in breast cancer tissues were both found to be higher compared with that of the clinical samples of normal adipose tissue or normal mammary tissue. Furthermore, the expression of PAI-1 in invasive breast cancer and PLOD2 expression in the corresponding peritumoral adipose tissue were positively correlated with each other (Fig. 7f-g). Taken together, these results showed that a higher amount of aligned collagen fibrils are deposited at the boundary between breast cancer and mammary adipose, and this phenomenon may be mediated by the high levels of tumor-derived PAI-1 and corresponding activation of PLOD2 in periphery adipose tissues (Fig. 7h).

## Discussion

The reciprocity between adipocytes and breast cancer cells has been studied for several years. Adipocytes play crucial roles in the proliferation, survival, and invasion of breast cancer cells via secreting cytokines (including leptin, adiponectin and IL-6) [15, 26] or providing energy (such as fatty acids) [11, 12]. During crosstalk with breast cancer cells, adipocytes undergo a dedifferentiation process and the shape of the adipocytes are phenotypically changed to resemble fibroblast-like cells, which is accompanied with increased secretion of fibronectin and collagen I [18]. However, the role of adipocyte-derived collagen in tumor progression has been given little attention, and the underlying biological mechanism of adipocyte-derived collagen is also poorly understood. Our proposed mechanism demonstrating the interaction between adipocytes and breast cancer cells promotes PLOD2-dependent adipocyte-derived collagen reorganization, which facilitates the migration of breast cancer along aligned, bundled collagen fibers is summarized in Fig. 7h. We showed that PLOD2 was upregulated in CAAs during.

adipocyte-derived collagen reorganization, inhibition of PLOD2 abrogated the linearly collagen deposited by CAAs, which further inhibited breast cancer metastasis. Further, we showed the secretion of PAI-1 by breast cancer cells exerted a key role in activating PLOD2 in CAAs through the PI3K/AKT-FOXP1 pathway. Moreover, increased linearity degree of collagen at the invasive front was observed in invasive breast cancer patients, and PLOD2 expression in peritumoral adipose tissue was positively correlated with the PAI-1 expression in the corresponding breast tumor tissue. Therefore, our study reveals a new stromal collagen network that favors tumor invasion and metastasis establish between breast cancer cells and surrounding adipocytes at the tumor invasive front.

Several studies have suggested that PLOD2 is correlated with poor prognosis of multiple cancers, including sarcoma, lung cancer, renal cell carcinoma, breast cancer, cervical cancer and bladder cancer [19, 27–31]. PLOD2 reinforces the migration capacity of cancer cells, and also promotes cancer cells migration along the “highway” linear collagen [19, 28, 32]. Previous studies showed silencing of PLOD2 led to the inhibition of migration and invasiveness of multiple cancer cells themselves, which might mediate by abrogating the epithelial-mesenchymal transition (EMT) of those cancer cells [33, 34]. In addition, the elevated PLOD2 expression in stromal cells promoted the alteration of collagen structure deposited by the stromal cells, which further enhanced cancer cells migration along the linearity collagen. In lung cancer, PLOD2 expression in CAFs resulted in the collagen contractile network formation and promoted tumor migration along the collagen network, while depletion of PLOD2 expression in CAFs diminished collagen network formation [35]. In pancreatic cancer, hypoxic pancreatic stellate cells (PSCs) high expression of PLOD2 to promote cancer cell motility through alteration of extracellular matrix (ECM) fiber architecture, while knockdown of PLOD2 in PSCs blocked parallel fiber architecture of matrices, leading to decreased directional migration of cancer cells within the matrices [36]. In our present study, PLOD2 was highly activated in CAAs, and pharmacological inhibition of PLOD2 abrogated the linear collagen deposition by CAAs (Fig. 1 and Fig. 2). In vivo subcutaneously adipose tissue and breast cancer cells co-injection metastatic model further confirmed the peritumoral adipose-derived collagen reorganization and PLOD2 was activated in adipocytes at the invasive front of breast cancer, adipose-breast cancer cells co-injection promoted tumor cells far away spread to lungs, while inhibition of PLOD2 inhibited breast cancer metastasis (Fig. 2 and Fig. 4). Mechanistic studies have revealed that PLOD2 can be regulated by numerous factors, including HIF-1α, TGF-β, EGF, miR 26a/b, IL-6 and leptin [9, 19, 27, 28, 32] . The paracrine signals derived from adipocytes or CAFs were reported to regulate PLOD2 expression in multiple cancers, suggesting the signals in microenvironment might involve in regulating PLOD2 expression. In breast cancer, PLOD2 was activated in breast cancer cells upon coculturing with adipocytes, while the knockdown of PLOD2 in cancer cells blocked the migration capacity stimulated by adipocytes [37]. Therefore, PLOD2 may be a potential therapeutic target of breast cancer, since impeding PLOD2 is able to directly inhibit cancer cell migration and indirectly block the role of stromal cell-derived collagen reorganization.

Upon secretion cytokine analysis, adipocyte-derived IL-6 or tumor-derived PAI-1 might exert roles in activating PLOD2 in CAAs (Fig. 3a-b). In breast cancer, the secretion of adipocyte-derived IL-6 is regulated by interaction with tumor cells [15]. The upregulation secretion of IL-6 by CAAs exerts multiple roles in cancer progression [10, 38, 39]. IL-6 has also been identified as a vital factor that is involved in collagen formation and fibrosis, which is mediated by regulating the JAK/STAT pathway and TGF-β1 [40]. It has been reported that adipocyte-derived IL-6 promoted the up-regulation of PLOD2 in breast cancer cells via activating JAK/STAT3 and PI3K/AKT pathways [37]. Nevertheless, in our present study, neutralization of IL-6 did not abrogate PLOD2 in CAAs, suggesting that IL-6 did not play a key role in inducing PLOD2 expression in CAAs (Fig. 3). Further studies showed PI3K/AKT pathway exerted the pivotal role in activating PLOD2 expression in CAAs, while the JAK/STAT3 pathway exhibited little function on regulating PLOD2 expression in CAAs (Fig. 5). Interestingly, neutralization of IL-6 significantly abrogated JAK/STAT3 pathway, while it exerted little role on the PI3K/AKT pathway in CAAs (results not shown), these results further explained the reason why IL-6 did not function in activating PLOD2 in CAAs, as the JAK/STAT3 axis was the main pathway for the activity of IL-6, but the JAK/STAT3 pathway played little effect on the regulation of PLOD2 expression in CAAs. These results indicating that unknown factors distinct from IL-6 mediated the induction of PLOD2 expression in CAAs.

PAI-1 has been shown to be indispensable for the proliferation, apoptosis, angiogenesis, metastasis of cancer and are significantly associated with poor prognosis in human cancers [41]. Notably, several studies suggested PAI-1 acted as an important factor involved in tumor metastasis. PAI-1 had been regarded as a mesenchymal marker and thoroughly confirmed to be a pivotal downstream effector on EMT induced by TGF-β in various cancer, such as lung cancer, gastric cancer and colorectal carcinoma cells [41–44]. It was also observed that PAI-1/PIAS3/Stat3/miR-34a feedback loop enhanced EMT-mediated metastasis through Stat3 signaling in non-small cell lung cancer (NSCLC) [45]. Furthermore, PAI-1 was reported to promote tumor metastasis via upregulating MMP13 expression and secretion in osteosarcoma [46]. PAI-1 secreted by metastatic ovarian cancer cells triggered the tumor-promoting role of the mesothelium in a feedback loop to accelerate ovarian cancer cells disseminated to peritoneum [47]. Moreover, in triple negative breast cancer (TNBC), PAI-1-secreted by TNBC cells could stimulate the expression and secretion of CCL5 from endothelial cells, which then enhanced TNBC cells migration, invasion, and metastasis [44]. Overexpression of PAI-1 in TNBC cells further promoted alignment of collagen perpendicular to the margin of tumor, suggesting PAI-1 might exert roles in surrounding tumor microenvironment in orthotopic breast cancer [48]. Interestingly, our present study indicated breast cancer cells secreted PAI-1 led to the activation of PLOD2 expression in adipocytes microenvironment, while the addition of tiplaxtinin further abrogated PLOD2 activation in CAAs (Fig. 3d-f). Knockdown of PAI-1 in breast cancer cells reversed the PLOD2 activation in CAAs, which subsequently inhibit breast cancer metastasis induced by adipose tissue in vivo. Hence, our study suggested PAI-1 may be a valuable target in breast cancer, targeting PAI-1 could inhibit cancer cells migration and abrogated PLOD2-mediated collagen reorganization in tumor microenvironment.

FOXP1 is involved in the transcriptional regulation and plays vital roles in immune responses, cell differentiation, and cancer progression [49, 50]. FOXP1 gradually decreases during adipogenesis, suggesting that FOXP1 is a negative regulator in the adipocytes differentiation process [51, 52] . Interestingly, we showed that FOXP1 was upregulated in adipocytes during adipocytes dedifferentiation, and was able to function as the transcription factor of PLOD2 in CAAs (Fig. 6). In addition, as FOXA1 plays a critical role in driving PLOD2 transcription expression in NSCLC [19], we also detected the role of FOXA1 in regulating PLOD2 in CAAs, and the results showed that the expression of FOXA1 was extremely low in adipocytes and was not consistently altered upon coculturing with different breast cancer cells (Additional file 5: Figure S3D). Therefore, FOXA1 does not function as the transcription factor in regulating PLOD2 activity in CAAs, while FOXP1 plays a key role in the transcription of PLOD2 in CAAs. These results suggest that the differences in the promoter sequence of PLOD2 may play a key role in determining the transcription regulator factors in different cells.

## Conclusion

Over the past several years, PLOD2 has been implicated as a protumorigenic agent in multiple cancers, particularly in cancer metastasis. We are the first to report that CAAs remodels collagen alignment in a PLOD2 dependent manner during crosstalk with breast cancer cells in vitro and in vivo, which further promotes breast cancer metastasis. We also demonstrate a novel role for PAI-1 in the induction of activating PLOD2 in CAAs through the PI3K/AKT-FOXP1 pathway. We anticipate that our findings will contribute to the development of new strategies for cancer treatment that involve targeting the tumor microenvironment, and targeting PLOD2 may be a credible therapeutic strategy for breast cancer metastasis via directly inhibiting cancer migration and indirectly blocking adipocyte-derived collagen reorganization.

## Additional files

Additional file 1: Supporting Information (DOC 28 kb)

Additional file 2: **Table S1.** Sequences of the primers used to detect genes expression by qRT-PCR. **Table S2.** Sequences of the primers used to detect genes expression by qRT-PCR. **Table S3.** The oligonucleotides of the PLOD2 promoter primers in CHIP assay. **Table S4.** The characteristics of patient samples (DOCX 22 kb)

Additional file 3: **Figure S1.** Breast cancer cells promote adipocyte-derived collagen I reorganization in vitro*.* (A) Analysis of protein expression of the indicated markers done by Western blots with extracts from mature adipocytes cocultivated with multiple breast cancer cells for 3 days. (B) The indicated proteins were measured in the ASCs adipocytes cultivated in the presence of MDA-MB-231 or SKBR-3 cells or in the absence of breast cancer cells for 72 h. (C) Immunofluorescence staining of type I collagen antibody (green) and DAPI (blue) of human adipocytes (control) or cocultivated adipocytes (co-culture). (d) Photographs showing collagen I matrix(3 mg/ml) contraction in the presence of either adipocytes, CAAs or breast cancer cells (TIF 1152 kb)

Additional file 4: **Figure S2.** PAI-1 induced PLOD2 activation in CAAs. (A) Immunoblot assay of adipocytes cultured alone or cocultured with SKBR-3 cells treated with 5 μg/μl IL-6 neutralizing antibody or tiplaxtinin (20 μM) for PLOD2 expression. (B) qPCR analysis of PLOD2 expression in adipocytes cultured alone or with breast cancer cells (MDA-MB-231 or SKBR-3) for 72 h. Data was normalized to 1 for monocultures using GAPDH as internal control. (D) mRNA expression of PLOD2 was analyzed after fed with PAI-1 recombinant protein. (E) Knockdown of PAI-1 in MDA-MB-231 cells. (F) Immunoblot assay of adipocytes cultured alone or cocultured with MDA-MB-231 cells or MDA-MB-231 shPAI-1 cells for PLOD2 expression. (G) Immunoblot assay of adipocytes cultured alone or stimulated with recombinant PAI-1 for LRP-1 expression (TIF 593 kb)

Additional file 5: **Figure S3.** FOXP1 acts as a transcription factor of PLOD2 in CAAs. (A) The effect of tiplaxtinin on the transcription of PLOD2 was evaluated by luciferase reporter assays. (B, C) The PAI-1 inhibitor (tiplaxtinin) decreased the expression of FOXP1 in CAAs. (D) Relative expression of FOXA1 in adipocytes cocutured with or without breast cancer cells (MDA-MB-231 or SKBR-3) (TIF 288 kb)

## Abbreviations

3D: Three-dimensional
ADF: Adipocyte-derived fibroblast
CAA: Cancer-associated adipocyte
CAF: Cancer-associated fibroblast
ECL: Enhanced Chemiluminescence
ECM: Extracellular matrix
ETP: Endotrophin
FOXP1: Forkhead box (FOX) P
FSP1: Fibroblast-specific protein
IGF-1: Insulin-like growth factor 1
IL-6: interleukin-6
LOX: L ysyl oxidase
MCP-1: Monocyte chemotactic protein-1
P4HA1: Prolyl 4-hydroxylase subunit alpha-1
PAI-1: Plasminogen Activator Inhibitor Type 1
PLOD2: Procollagen-lysine, 2-oxoglutarate 5-dioxygenase 2
TIMP1: Tissue inhibitor of metalloproteinase 1
TNF-α: Tumor necrosis factor
