# Supplementary material for: Tumor-secreted PAI-1 promotes breast cancer metastasis via the induction of adipocyte-derived collagen remodeling
Source: Cell Commun Signal. 2019 Jun 6;17:58. doi: 10.1186/s12964-019-0373-z (PMC6554964; doi:10.1186/s12964-019-0373-z)
Supplement: Supplementary file 1 — Supporting Information (DOC 28 kb) [file 12964_2019_373_MOESM1_ESM.doc]

Supporting Information

**Materials and Methods**

**Antibodies and reagents**

All the antibodies were purchased from Cell Signaling Technology, R&D Systems or Abcam. Anti-C/EBPα, anti-FSP1, anti-α-SMA, anti- PAI-1, anti-AKT, anti-phospho-Akt (Ser473), anti-STAT3, anti-phospho-STAT3(Tyr705), anti-FOXP1, anti-Histon H3, anti-GAPDH, and secondary antibodies were purchased from Cell Signaling Technology. Anti-PLOD2 (21214-1-AP) was purchased from Proteintech and anti- PLOD2 (MAB4445) was purchased from R&D Systems, anti-collagen I was purchased from Abcam, anti-LOX was purchased from Abclone. Goat anti-mouse IgG (H+L) Secondary antibody, Alexa Fluor® 488 conjugate and goat anti-rabbit IgG (H+L) Secondary antibody, Alexa Fluor® 594 conjugate were from Thermo Fisher Scientific. The recombinant PAI-1 was from Pepro Tech, recombinant IL-6 a, IL-6 neutralizing antibody and RAP were purchased from R&D Systems. Tiplaxtinin was purchased from A dooq, LY294002 and ruxolitinib were purchased from Selleckchem.

**Western blotting**

The total cell lysates were extracted from the untreated/treated cells, adding phosphatase and protease inhibitors. The proteins were fractionated by 6%-15% sodium dodecyl sulfate polyacrylamide gel electrophoresis (SDS-PAGE) and electro-blotted onto PVDF membrane (Millipore, USA). Next, the membranes were blocked with 5% BSA and incubated with primary antibodies (dilution in 1% BSA-TBST) for overnight at 4°C. Then, probed it with secondary antibody for 1h at room temperature. Subsequently, the expression of the target proteins was detected by Chemiluminescent HRP Substrate (Millipore, USA).

**Immunofluorescence assay**

Adipocytes differentiated on coverslips grown alone, or cocultivated for the indicated times with breast cancer cells were processed for immunofluorescence. Cells were fixed with 4% paraformaldehyde and blocked with PBS supplemented with 10% FBS for 20 min. Fluorescent antibody against collagen I (Abcam) and DAPI (Invitrogen) were used for immunofluorescence. Fluorescence images were acquired with a confocal laser microscopy system (Olympus). For each sample, more than 100 cells were examined in at least three independent experiments.

**Immunohistochemistry (IHC) assay**

Immunohistochemistry was conducted as previously. Breifly, the tissue sections were deparaffinized and immersed for 10 min in PBS. After antigen retrieval, the endogenous peroxidase was blocked by 3% H_2_O_2_, and non-specific staining was blocked by 5% goat serum. The sections were subsequently incubated with primary antibodies overnight at 4°C then treated with biotinylated secondary antibody for 30 minutes respectively. The sections were then exposed to DAB for 6 min. The evaluation of the IHC staining was performed by pathologist.
