# Supplementary material for: Tumor-secreted PAI-1 promotes breast cancer metastasis via the induction of adipocyte-derived collagen remodeling
Source: Cell Commun Signal. 2019 Jun 6;17:58. doi: 10.1186/s12964-019-0373-z (PMC6554964; doi:10.1186/s12964-019-0373-z)
Supplement: Supplementary file 2 — Table S1. Sequences of the primers used to detect genes expression by qRT-PCR. Table S2. Sequences of the primers used to detect genes expression by qRT-PCR. Table S3. The oligonucleotides of the PLOD2 promoter primers in CHIP assay. Table S4. The characteristics of patient samples (DOCX 22 kb) [file 12964_2019_373_MOESM2_ESM.docx]

Table S1. Sequences of the primers used to detect genes expression by qRT-PCR

| **Name** | **Forward primer** | **Reverse primer** |
| --- | --- | --- |
| m-GAPDH | CATCACTGCCACCCAGAAGACTG | ATGCCAGTGAGCTTCCCGTTCAG |
| m-PLOD2 | CATCCGAGAGTTCATTGCTCCAG | GCGCTGTCTTTCAGGTGAGTAC |
| m-LOX | CATCGGACTTCTTACCAAGCCG | GGCATCAAGCAGGTCATAGTGG |
| m-COL1A1 | CCTCAGGGTATTGCTGGACAAC | CAGAAGGACCTTGTTTGCCAGG |
| m-IL-4 | ATCATCGGCATTTTGAACGAGGTC | ACCTTGGAAGCCCTACAGACGA |
| m-TNF RII  m-OPG | TGACAGGAAGGCTCAGATGTGC  CGGAAACAGAGAAGCCACGCAA | ATGCTTGCCTCACAGTCCGCAC  CTGTCCACCAAAACACTCAGCC |
| m-MIF  m-TIMP2  m-IGFBP2  m-ANGPTL-4  m-TECK  m-IFN-γ  m-FGF-6  m-TNFα  m-PDGF-AA  m-TIMP1  m-MCP-1  m-Leptin  m-IL-6  m-IL-6 sR  h-IGFBP1  h-IFNɣ  h-MCP-1  h-OPG  h-IL-6 sR  h-IL-6  h-IL-1β  h-IL-1α  h-IL-8  h-SDF-1  h-FGF-6  h-TECK  h-RANTES  h-ENA78  h-IGFBP2  h-PAI-1  h-IL-11  h-MIP-1β  h- TNF RII | GAACCGCAACTACAGTAAGCTGC  AGCCAAAGCAGTGAGCGAGAAG  CCTCAAGTCAGGCATGAAGGAG  CTGGACAGTGATTCAGAGACGC  AAGGCTAGTCCACTGGAAGAGC  CAGCAACAGCAAGGCGAAAAAGG  GCATCAGTGGAACACACGAGGA  GGTGCCTATGTCTCAGCCTCTT  CTGGCTCGAAGTCAGATCCACA  TCTTGGTTCCCTGGCGTACTCT  GCTACAAGAGGATCACCAGCAG  GCAGTGCCTATCCAGAAAGTCC  TACCACTTCACAAGTCGGAGGC  TGCAGTTCCAGCTTCGATACCG  TCCTTTGGGACGCCATCAGTAC  GAGTGTGGAGACCATCAAGGAAG  AGAATCACCAGCAGCAAGTGTCC  GGTCTCCTGCTAACTCAGAAAGG  GACTGTGCACTTGCTGGTGGAT  AGACAGCCACTCACCTCTTCAG  CCACAGACCTTCCAGGAGAATG  TGTATGTGACTGCCCAAGATGAAG  GAGAGTGATTGAGAGTGGACCAC  CTCAACACTCCAAACTGTGCCC  AGGCGTGGTGAGTCTCTTTGGA  TGTCTTTGAGGACTGCTGCCTG  CCTGCTGCTTTGCCTACATTGC  CAGACCACGCAAGGAGTTCATC  CGAGGGCACTTGTGAGAAGCG  CTCATCAGCCACTGGAAAGGCA  GGACCACAACCTGGATTCCCTG  GCTTCCTCGCAACTTTGTGGTAG  CCGCTTCAGAAAACCACCTCAG | ACGTTGGCAGCGTTCATGTCGT  GCCGTGTAGATAAACTCGATGTC  TGGTCCAACTCCTGCTGGCAAG  GATGCTGTGCATCTTTTCCAGGC  GTGGCACTCCTCACGCTTGTAC  TTTCCGCTTCCTGAGGCTGGAT  CAGTCTTCCTTTACTGTTCATGGC  GCCATAGAACTGATGAGAGGGAG  GACTTGTCTCCAAGGCATCCTC  GTGAGTGTCACTCTCCAGTTTGC  GTCTGGACCCATTCCTTCTTGG  GGAATGAAGTCCAAGCCAGTGAC  CTGCAAGTGCATCATCGTTGTTC  TGCTTCACTCCTCGCAAGGCAT  GATGTCTCCTGTGCCTTGGCTA  TGCTTTGCGTTGGACATTCAAGTC  TCCTGAACCCACTTCTGCTTGG  CAGCAAACCTGAAGAATGCCTCC  ACTTCCTCACCAAGAGCACAGC  TTCTGCCAGTGCCTCTTTGCTG  GTGCAGTTCAGTGATCGTACAGG  AGAGGAGGTTGGTCTCACTACC  CACAACCCTCTGCACCCAGTTT  CTCCAGGTACTCCTGAATCCAC  TTGTTGGGCAGGAGGGTTTCTC  ACACCTTCCTGTGTCTCTTGGG  ACACACTTGGCGGTTCTTTCGG TTCCTTCCCGTTCTTCAGGGAG  TGTTCATGGTGCTGTCCACGTG GACTCGTGAAGTCAGCCTGAAAC  AGTAGGTCCGCTCGCAGCCTT  GGTCATACACGTACTCCTGGAC  ATGCCGGTACTGGTTCTTCCTG |

Table S2. Sequences of the primers used to detect genes expression by qRT-PCR.

| **Name** | **Forward primer** | | **Reverse primer** |
| --- | --- | --- | --- |
| m-Nrf1 | | AGAACGAGAGGACACCTGGTCA | GCTTCTGGGATGCTGGAAACGT |
| m-AXUD1 | | TGCCTGGAACTCTGATGAGGAG | AGACGGTGATGCCGTTAAAGGC |
| m-FOXP1 | | CATGCCTCTACCAATGGACAGC | GAAGTCGTCACAAACCGCCTCA |
| m-FOXH1  m-FOXO1  m-FOXO3a  m-RUNX3 | | ACTATGAGGGCTGGAAGGACTC  CTACGAGTGGATGGTGAAGAGC  CCTACTTCAAGGATAAGGGCGAC  CAGGTTCAACGACCTTCGATTCG | AGGAATCAGGCTCACATCCACC  CCAGTTCCTTCATTCTGCACTCG  GCCTTCATTCTGAACGCGCATG  TCCACAGTGACCTTGATGGCTC |

Table S3. The oligonucleotides of the PLOD2 promoter primers in CHIP assay.

| **Promoter Primers** | **Forward primer** | **Reverse primer** |
| --- | --- | --- |
| primer 1 | TGGAACCCCGGGAGAGCAGAGG | GGCGGGGCCAGGGCCGGGGG |
| primer 2 | CTCCTGGCACCAGCAGCGGCAG | GACTGCGAGGGCTTCAGTCTGC |
| primer 3 | AGCAGTGGAGGTAGCTCCAGG | AGCCATAGACTCTTCCAGTAATG |
| primer 4 | TCTTGAACGCGGTCAATTTGAT | TTGGGTTTTGATTAGTATGTCTAT |
| primer 5 | TACACATTTTCCCACCTGCTTT | AGCTCGTAGTTAAATCTTCCTG |
| primer 6  primer 7 | TGCAATCACAGTTTCCATTTCT  AAACATATTTAGCCAAATGACC | AATATTAGGAGTGTGACATTTAAA  GAAGAATGGAGTAAGGAAGAAT |
| primer 8  primer 9  primer10  primer11  primer12 | GCCTTTGAGACTCAACAGACTG  AGCTGAGTATTTGCCATGGGAA  GAAAGAATGAATGGAAGAAGGC  TTCCGGTTCTGTGGAATCTTTG  GTAGAGGAAAGGAGAAGAGAGT | CCAGAATCCAGACTTGGGCCT  AGAACTTAAGAGCCTCTATAAA  TCTTTAGCTCATCAAATCCAGA  CTGCTTAAGTAACATACCTCTT  ACAATCCTAACCCATTTGCCTA |

| **Characteristics** | **Sub-groups** | **Number** |
| --- | --- | --- |
| Age (years) | < 50 | 3 |
|  | ≥50 | 13 |
| Tumor size (cm) | <2 | 5 |
|  | ≥2 | 11 |
| TNM stage | I-II | 10 |
|  | III-IV | 6 |
| ER status | Negative | 9 |
|  | Positive | 7 |
| PR status | Negative | 7 |
|  | Positive | 9 |
| HER-2 status | Negative | 8 |
|  | Positive | 8 |
| Lymph nodal status | Negative | 6 |
|  | Positive | 10 |

Table S4. The characteristics of patient samples.
