# Supplementary figures and images for: Tumor-secreted PAI-1 promotes breast cancer metastasis via the induction of adipocyte-derived collagen remodeling
Source: Cell Commun Signal. 2019 Jun 6;17:58. doi: 10.1186/s12964-019-0373-z (PMC6554964; doi:10.1186/s12964-019-0373-z)

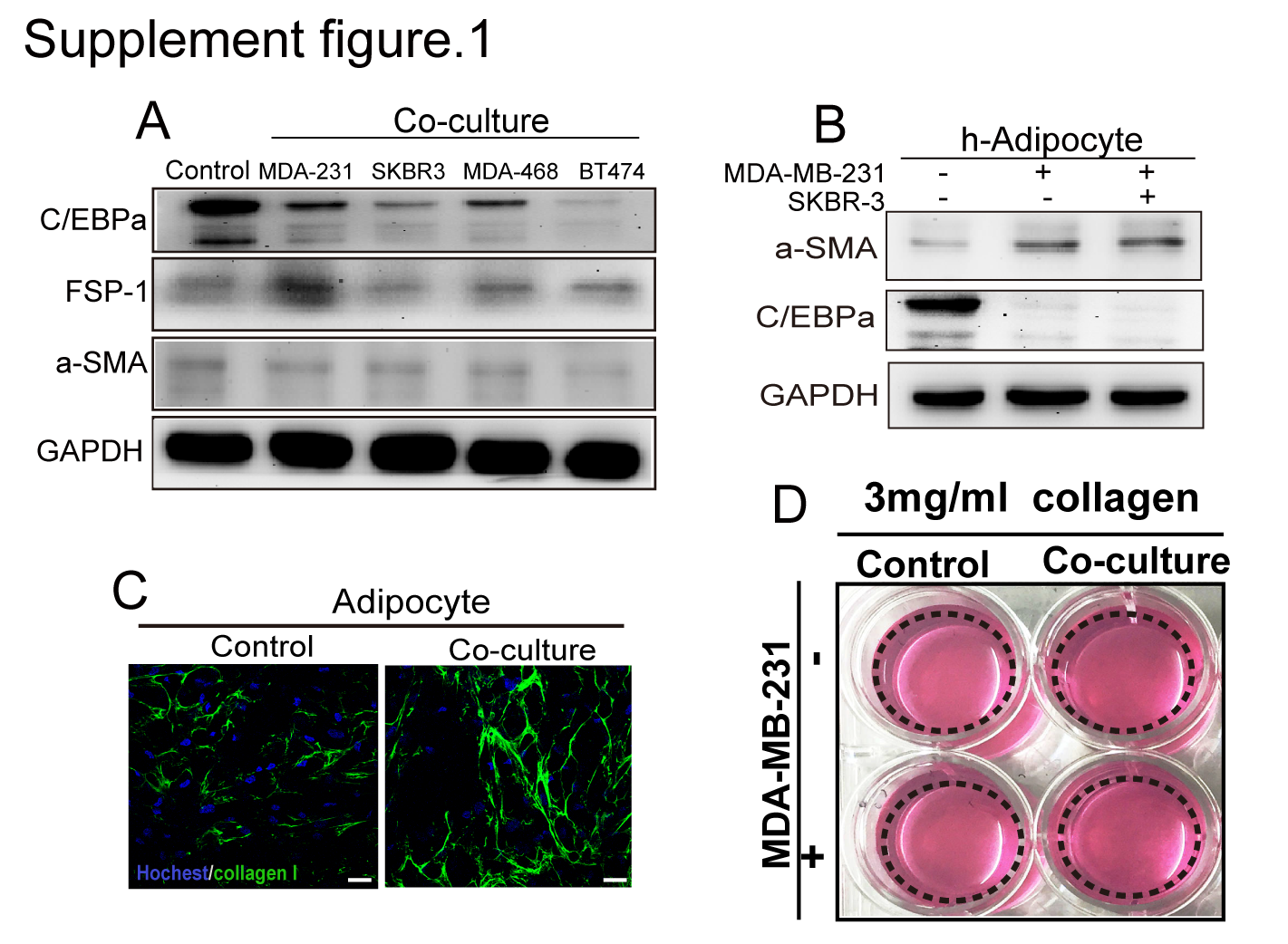

Supplement: Supplementary file 3 — Figure S1. Breast cancer cells promote adipocyte-derived collagen I reorganization in vitro. (A) Analysis of protein expression of the indicated markers done by Western blots with extracts from mature adipocytes cocultivated with multiple breast cancer cells for 3 days. (B) The indicated proteins were measured in the ASCs adipocytes cultivated in the presence of MDA-MB-231 or SKBR-3 cells or in the absence of breast cancer cells for 72 h. (C) Immunofluorescence staining of type I collagen antibody (green) and DAPI (blue) of human adipocytes (control) or cocultivated adipocytes (co-culture). (d) Photographs showing collagen I matrix(3 mg/ml) contraction in the presence of either adipocytes, CAAs or breast cancer cells (TIF 1152 kb) [file 12964_2019_373_MOESM3_ESM.tif]

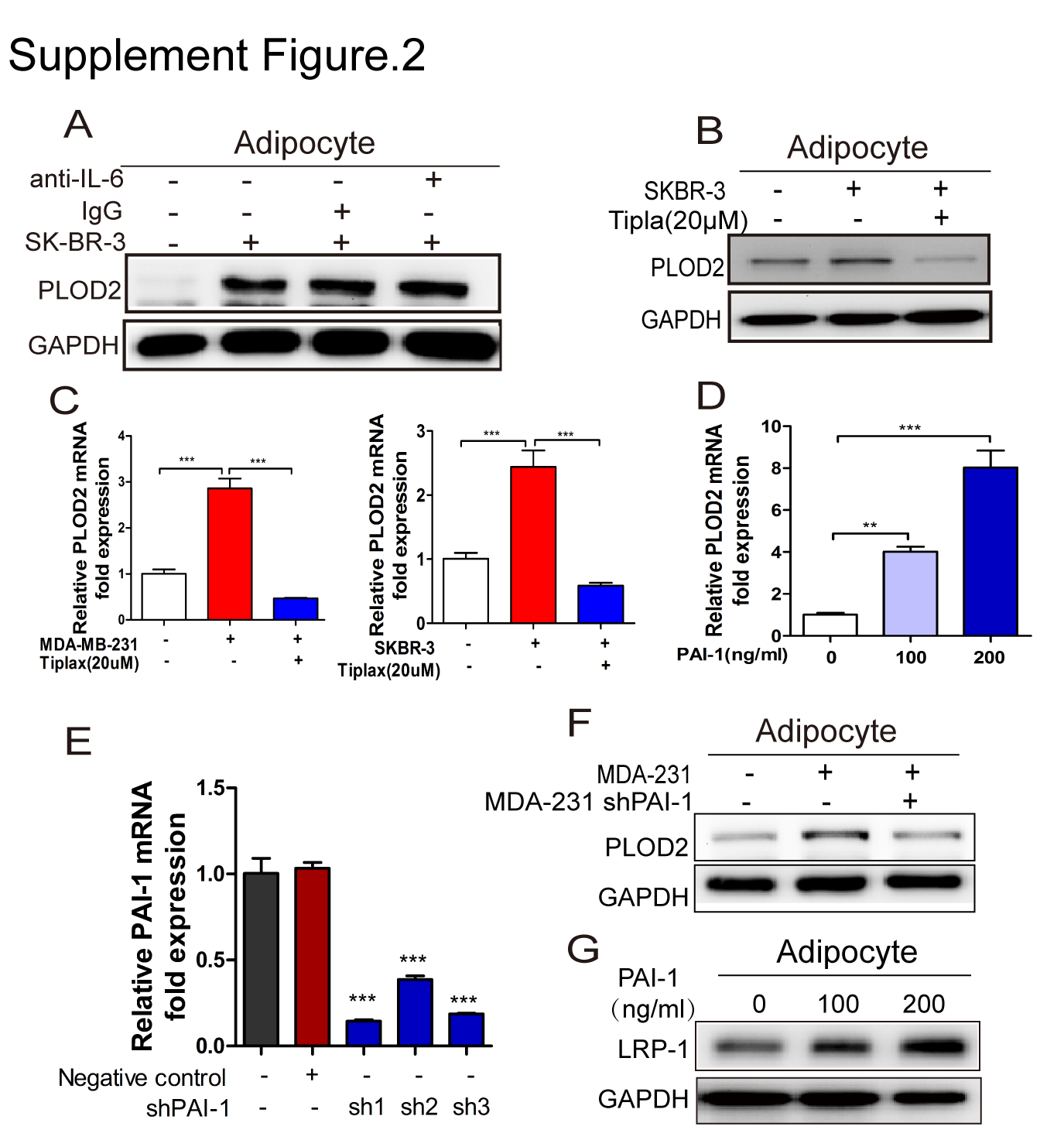

Supplement: Supplementary file 4 — Figure S2. PAI-1 induced PLOD2 activation in CAAs. (A) Immunoblot assay of adipocytes cultured alone or cocultured with SKBR-3 cells treated with 5 μg/μl IL-6 neutralizing antibody or tiplaxtinin (20 μM) for PLOD2 expression. (B) qPCR analysis of PLOD2 expression in adipocytes cultured alone or with breast cancer cells (MDA-MB-231 or SKBR-3) for 72 h. Data was normalized to 1 for monocultures using GAPDH as internal control. (D) mRNA expression of PLOD2 was analyzed after fed with PAI-1 recombinant protein. (E) Knockdown of PAI-1 in MDA-MB-231 cells. (F) Immunoblot assay of adipocytes cultured alone or cocultured with MDA-MB-231 cells or MDA-MB-231 shPAI-1 cells for PLOD2 expression. (G) Immunoblot assay of adipocytes cultured alone or stimulated with recombinant PAI-1 for LRP-1 expression (TIF 593 kb) [file 12964_2019_373_MOESM4_ESM.tif]

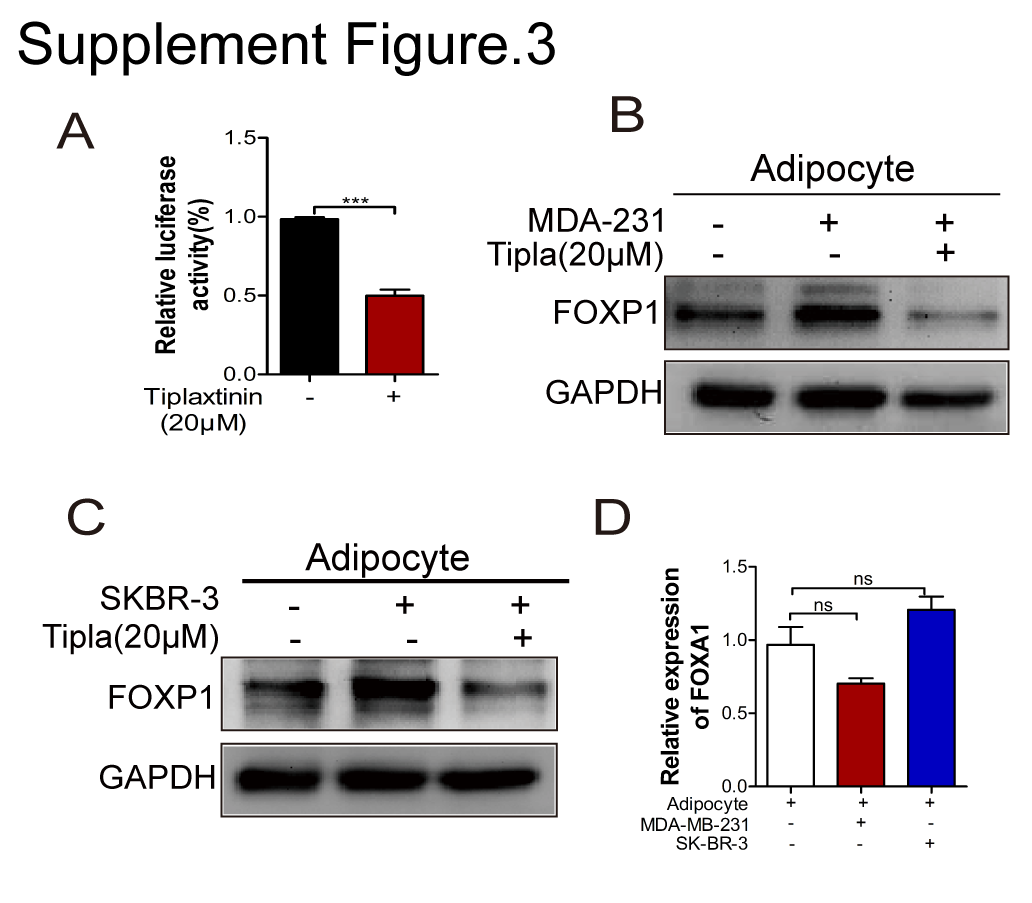

Supplement: Supplementary file 5 — Figure S3. FOXP1 acts as a transcription factor of PLOD2 in CAAs. (A) The effect of tiplaxtinin on the transcription of PLOD2 was evaluated by luciferase reporter assays. (B, C) The PAI-1 inhibitor (tiplaxtinin) decreased the expression of FOXP1 in CAAs. (D) Relative expression of FOXA1 in adipocytes cocutured with or without breast cancer cells (MDA-MB-231 or SKBR-3) (TIF 288 kb) [file 12964_2019_373_MOESM5_ESM.tif]
